# Collagen IV deficiency causes hypertrophic remodeling and endothelium-dependent hyperpolarization in small vessel disease with intracerebral hemorrhage

**DOI:** 10.1016/j.ebiom.2024.105315

**Published:** 2024-08-30

**Authors:** Sarah McNeilly, Cameron R. Thomson, Laura Gonzalez-Trueba, Yuan Yan Sin, Alessandra Granata, Graham Hamilton, Michelle Lee, Erin Boland, John D. McClure, Cristina Lumbreras-Perales, Alisha Aman, Apoorva A. Kumar, Marco Cantini, Caglar Gök, Delyth Graham, Yasuko Tomono, Christopher D. Anderson, Yinhui Lu, Colin Smith, Hugh S. Markus, Marc Abramowicz, Catheline Vilain, Rustam Al-Shahi Salman, Manuel Salmeron-Sanchez, Atticus H. Hainsworth, William Fuller, Karl E. Kadler, Neil J. Bulleid, Tom Van Agtmael

**Affiliations:** a School of Cardiovascular and Metabolic Health, College of Medical Veterinary and Life Sciences, University of Glasgow, Glasgow G12 8QQ, UK; b Department of Clinical Neurosciences, Victor Phillip Dahdaleh Heart and Lung Research Institute, University of Cambridge and Royal Papworth Hospital, Cambridge, UK; c Glasgow Polyomics, University of Glasgow, Glasgow, UK; d School of Health and Wellbeing, College of Medical Veterinary and Life Sciences, University of Glasgow, Glasgow, UK; e Molecular and Clinical Sciences Research Institute, St George’s University of London, London, UK; f Princess Royal University Hospital, Kings College Hospital NHS Foundation Trust, London, UK; g Centre for the Cellular Microenvironment, School of Science and Engineering, University of Glasgow, Glasgow, UK; h Division of Molecular & Cell Biology, Shigei Medical Research Institute, Okayama, Japan; i Department of Neurology, Brigham and Women’s Hospital, Boston, MA, USA; j Wellcome Centre for Cell Matrix Research, Faculty of Biology, Medicine & Health, University of Manchester, Manchester, UK; k Academic Neuropathology, University of Edinburgh, Edinburgh, UK; l Department of Neurology, Cambridge University Hospitals NHS Foundation Trust, Cambridge, UK; m Department of Genetics, Hôpital Erasme, ULB Center of Human Genetics, Universite Libre de Bruxelles, Bruxelles, Belgium; n Centre for Clinical Brain Sciences, University of Edinburgh, Edinburgh, UK; o School of Molecular Biosciences, College of Medical Veterinary and Life Sciences, University of Glasgow, Glasgow, UK; p Center for Genomic Medicine, Massachusetts General Hospital, Boston, MA, USA

**Keywords:** Collagen, Basement membrane, Cerebrovascular disease, Stroke, Small vessel disease, Endothelial dysfunction

## Abstract

**Background:**

Genetic variants in *COL4A1* and *COL4A2* (encoding collagen IV alpha chain 1/2) occur in genetic and sporadic forms of cerebral small vessel disease (CSVD), a leading cause of stroke, dementia and intracerebral haemorrhage (ICH). However, the molecular mechanisms of CSVD with ICH and *COL4A1/COL4A2* variants remain obscure.

**Methods:**

Vascular function and molecular investigations in mice with a *Col4a1* missense mutation and heterozygous *Col4a2* knock-out mice were combined with analysis of human brain endothelial cells harboring *COL4A1/COL4A2* mutations, and brain tissue of patients with sporadic CSVD with ICH.

**Findings:**

*Col4a1* missense mutations cause early-onset CSVD independent of hypertension, with enhanced vasodilation of small arteries due to endothelial dysfunction, vascular wall thickening and reduced stiffness. Mechanistically, the early-onset dysregulated endothelium-dependent hyperpolarization (EDH) is due to reduced collagen IV levels with elevated activity and levels of endothelial Ca^2+^-sensitive K^+^ channels. This results in vasodilation via the Na/K pump in vascular smooth muscle cells. Our data support this endothelial dysfunction preceding development of CSVD-associated ICH is due to increased cytoplasmic Ca^2+^ levels in endothelial cells. Moreover, cerebral blood vessels of patients with sporadic CSVD show genotype-dependent mechanisms with wall thickening and lower collagen IV levels in those harboring common non-coding *COL4A1/COL4A2* risk alleles.

**Interpretation:**

*COL4A1/COL4A2* variants act in genetic and sporadic CSVD with ICH via dysregulated EDH, and altered vascular wall thickness and biomechanics due to lower collagen IV levels and/or mutant collagen IV secretion. These data highlight EDH and collagen IV levels as potential treatment targets.

**Funding:**

MRC, Wellcome Trust, 10.13039/501100000274BHF.

**Research in context**

**Evidence before this study**

Cerebral small vessel disease (CSVD) causes most strokes due to brain bleeding (intracerebral haemorrhage, ICH) and is the major cause of dementia due to vascular disease accounting for ∼50% of all dementia cases. The genes *COL4A1/COL4A2* encode a collagen IV protein that is a major component of the basement membrane, a specialised extracellular matrix structure, in blood vessels. Previous studies showed that mutations in these genes cause a rare genetic form of CSVD as part of COL4A1-Syndrome (Gould Syndrome). In addition, for late-onset sporadic CSVD non-coding variants are a risk factor in 65% of the population, while rare coding variants also occur. However, the molecular mechanisms causing CSVD and of genetic variants in *COL4A1/COL4A2* remain obscure.

**Added value of this study**

We uncovered that collagen IV regulates endothelial cell mediated vasodilation of small arteries and that CSVD encompasses increased vasodilation with dysregulated endothelial hyperpolarization, providing new insight into mechanisms of CSVD in mice. This vascular dysfunction is accompanied by reduced vascular stiffness and increased wall thickness, and represents an early CSVD disease stage before development of ICH. This is driven by basement membrane alterations due to reduced levels of collagen IV, which cause late-onset micro-ICH and endothelial dysfunction. This mechanism is supported by data from our analysis of brain tissue from people with sporadic CSVD, which also provides new insight into a genotype-phenotype correlation of this disease. Analysis of cerebral blood vessels from patients who did not have rare coding *COL4A1/COL4A2* variants indicates common non-coding risk variants act by reduced collagen IV levels and increased vascular wall thickness. This wall thickening also occurs in people with CSVD carrying rare *coding COL4A1/COL4A2* variants, with data suggesting they act via secreting mutant protein, and thus represents a point where the mechanisms of these different genetic variants converge.

**Implications of all the available evidence**

These data create new insight into the regulation of vascular function and mechanisms of CSVD. They establish that increased EDH vasodilation occurs in early stages of CSVD, driven by reduced extracellular collagen IV levels that can be due to rare coding or common non-coding *COL4A1/COL4A2* variants and mutations. The finding of excessive EDH vasodilation via reduced extracellular collagen IV levels provides new targets for future treatment strategies.

## Introduction

Cerebral small vessel disease (CSVD) affects the arterioles, capillaries and venules of 80% of adults aged ≥ 65,^1^ and is a leading cause of stroke, dementia and intracerebral haemorrhage (ICH),^2^ contributing to ∼50% of dementia cases.^3^ A limited understanding of its molecular mechanisms hinders the ability to address the unmet need for treatments. In blood vessels a basement membrane (BM), a specialised extracellular matrix (ECM) structure, separates endothelial cells from vascular smooth muscle cells (VSMC), and collagen IV proteins form a network in BMs.^4^ The vascular BM contains a collagen IV network of α1α1α2(IV) proteins consisting of two α1(IV) chains and one α2(IV) chain, encoded by the genes *COL4A1* and *COL4A2*.^5^ Mutations in *COL4A1*/*COL4A2* cause early-onset CSVD with ICH, eye, muscle and kidney defects as part of the multi-systemic COL4A1 syndrome (referred to as Gould Syndrome).^5^ Common non-coding variants, that likely affect expression levels, and rare coding variants in *COL4A1*/*COL4A2* occur in ∼65% and 3% of sporadic CSVD,^6^, ^7^, ^8^ respectively, establishing collagen IV as a key genetic determinant.

BM defects, collagen IV misfolding with ER retention and ER stress can occur due to *COL4A1/COL4A2* mutations^5^^,^^9^, ^10^, ^11^ but the molecular cross-talk and relative contribution of these upstream molecular insults to the pathologies in Gould syndrome remains unknown. This is compounded by a lack of insight into the nature of the vascular defects in CSVD and how collagen IV mutations cause them.

Vascular dysfunction in CSVD is typified by reduced endothelial cell mediated vasodilation and associated hypoperfusion^12^ that correlates with CSVD severity.^13^ The three main endothelium-dependent vasodilatory pathways are 1) nitric oxide (NO) generation by NOS (NO synthase) leading to cGMP production in VSMCs; 2) prostacyclin production from cyclooxygenase; and 3) activation of calcium ion-dependent potassium channels (K_Ca_) in endothelial cells result in EDH in VSMC hyperpolarization and vasorelaxation. While these pathways are well-understood, the role of the ECM and BM therein remains underexplored. Consequently, whether *COL4A1/COL4A2* mutations affect endothelium-dependent vasodilation of small blood vessels is an important gap in our knowledge that is key to understanding CSVD.

Here we show collagen IV is a regulator of endothelial cell-mediated vasodilation and that in CSVD *Col4a1* missense mutations cause endothelial dysfunction with dysregulated EDH vasodilation through reduced extracellular collagen IV levels, and biomechanical and structural vascular defects. Our data also provide evidence that common non-coding variants and rare coding *COL4A1/COL4A2* variants exhibit genotype-dependent mechanisms that converge on hypertrophic remodeling of cerebral blood vessels. The identification of excessive EDH vasodilation and lower collagen IV levels represents a new mechanism in CSVD that highlights putative treatment avenues.

## Methods

### Ethics

Animal studies were performed in accordance with UK Home Office regulations (Project licenses 70/8604, PP9995833) and the University of Glasgow Animal Welfare and Ethics Review Board. We selected post-mortem paraffin-embedded human brain samples from the UK MRC Brain Bank (University of Edinburgh) and LINCHPIN study (NHS Scotland Research Ethics Committee, 10/MRE00/23).^2^^,^^14^ Brain tissue was collected with informed consent from patients or their families. Cell culture experiments were approved by the local Ethics Committees at the University of Glasgow (200200029) and Cambridge University (Ethics REC NO 16/EE/0118). HIPSC cell lines were established from skin biopsies following informed consent.^15^

### Animal studies

Mice were housed in open top cages containing bedding, nest building material and a shelter in a 12-h light/dark cycle with free access to food and water. Health monitoring of animals was performed daily. *Col4a1*^*+/SVC*^, *Col4a2*^*+/em2Wtsi*^ and wild type littermates (C57BL6/J genetic background) were randomly allocated to cohorts. Cohorts contained male and female mice as no data have been published indicating sex effects on ICH in *Col4a1* mutant mice. There were no criteria used for inclusion or exclusion of animals. In this study, ICH was considered a primary outcome and n = 6 per group is sufficient to have 80% power to find a difference in proportions between groups of 0.74 or more with a Type I error rate of 5%. Tissues were harvested between 9 am and 11 am to account for any influence of circadian rhythm. Cage location and order of analysis of mice was random. Samples were labelled numerically without genotype status, blinding the researcher. Unblinding occurred following completion of datasets. Animals were sacrificed using an increasing gradient of CO_2_, cervical dislocation, or with an isopentobarbitol intraperitoneal injection prior to 4% PFA perfusion fixation.

### Wire myography

Artery rings were prepared from third order mesenteric arteries and mounted on a myograph (DMT) in PSS. Vessels were normalised by stretching to a tension of 13.3 kPA and subjected to a wake-up procedure with KPSS (PSS plus 62.5 mM KCl). Vasoconstriction was measured by adding noradrenaline. Endothelium-dependent vasodilation was measured using dose–response curve of pre-constricted vessels (3 × 10^−6^ M noradrenaline) to carbachol (1 × 10^−8^ − 3 × 10^−5^ M). Contribution of NO was assessed by incubating vessels with 100 μM L-NAME (30 min) followed by carbachol dose response curve. Basal NO levels were measured indirectly by contracting vessels with 10 μM noradrenaline with/without prior L-NAME treatment. ΔmN = mN (L-NAME) - mN (untreated). To assess EDH-vasodilation, vessels were incubated with 1 μM TRAM-34 and 0.5 μM apamin for 20 min, followed by a carbachol dose response curve. The role of the Na^+^/K^+^ pump was measured by pre-treating arteries with 10 μM ouabain, followed by carbachol dose response curve in presence of L-NAME. Prostacyclin-mediated vasodilation was measured by carbachol dose response curve in presence of 10 μM indomethacin (cyclooxygenase inhibitor). Endothelium independent relaxation was measured using NO-donor sodium nitroprusside (SNP).

**Vascular structure and stiffness** were measured using a pressure myograph system as described^16^ (DMT). Vessels subjected to a pressure curve under passive conditions in PSS. Internal (Di) and external diameter (De) were measured after 5 min at each pressure. Wall thickness was calculated as [(De − Di)/2]. Cross sectional area CSA was calculated as [(π/4) × (De^2^−Di^2^)]. Wall lumen ratio W:L was calculated as [(De-Di)2∗Di]. Circumferential wall stress σ was calculated as [(P∗Di)/(2∗*wall thickness*)] whereby P = Pressure. Circumferential wall strain ε was calculated as [(Di–Di@10 mmHg)/(Di@10 mmHg). Overall stiffness β can be calculated as [ln (P/Ps) = β(De/Ds-1)], where Ps and Ds are the reference pressure and external diameter at the reference pressure (80 mm Hg).^17^

### Blood pressure analysis

Blood pressures were measured in *Col4a2*^*+/em2Wtsi*^ and WT littermates via radiotelemetry as described.^18^ PhysioTel PA-C10 pressure transmitters (DSI, St Paul, MN) were implanted with catheter inserted in the left carotid artery and transmitter on the shoulder. Surgery was performed under aseptic conditions and anaesthesia (2.5% isoflurane, 1.5 L/min O_2_). Analgesics (5 mg/kg carprofen) were administered post-surgery and mice recovered in a heated incubation box until fully conscious and monitored daily. Blood pressure was recorded one week after surgery. Measurements were recorded every 5 min for 48 h using the Dataquest IV Telemetry System (DSI). Day/night means were calculated based on 12-h light/dark cycle. Systolic blood pressure of 4–6-month-old *Col4a1*^*+/SVC*^ was recorded using tail plethysmography (IITC model 129 analyser, Woodland Hills, CA, USA) as described.^10^ Data were generated from the average of 4 readings taken on three independent occasions per animal, following three periods of training.

**Metabolic cage** and **slit lamp** (Righton) analysis was performed as previously described.^11^^,^^19^^,^^20^

### Histopathology of mouse tissue

Tissues were perfusion fixed in 4% PFA, followed by 24 h fixation (4% PFA). Paraffin embedded mid brains were serial sectioned (5–7 μm) covering a distance of ∼1 mm. Perl’s Prussian Blue (1% HCL, 1% Potassium ferrocyanide trihydrate (Acros Organics)) staining was used to determine ICH^11^ by staining for 30 min, washed in dH_2_0, counterstaining with Nuclear Fast Red (Acros Organics) and de-staining in tap water. Kidney sections were stained with PAS stain. Briefly, sections were submerged in 1% Periodic Acid (Sigma) for 5 min, followed by 5 min wash under running tap water, incubation with Schiff’s reagent (20 min, Fisher Scientific). After washing (5 min) under running tap water, they were counterstained with Harris’ Heamatoxylin and washed under running tap water (5 min). Stained sections were mounted using DPX (Sigma–Aldrich).

**Immunostaining of mouse tissue** was performed as described.^10^^,^^11^ Tissues were frozen in OCT on liquid nitrogen. Cryosections were fixed with acetone, incubated with 0.01 M HCl/KCl (antigen retrieval). Following blocking (1 h, 10% goat serum in PBS) sections were incubated with primary antibodies (Overnight, 4 °C, Supplemental Table S2), washed in PBS, and incubated (1 h) with secondary antibodies (Supplemental Table S2). Slides were mounted using VECTASHIELD Antifade Mounting Medium with DAPI (Vectorlabs). Images were obtained on Nikon Eclipse Ts2 microscope with DS-Fi3 camera and captured using NIS Elements software (Nikon).

**Electron Microscopy** was performed as described.^21^^,^^22^ Tissues were fixed in 1% osmium tetroxide (w/v) and 1.5% potassium ferrocyanide (w/v) in 0.1 M sodium cacodylate buffer (pH 7.2) for 1 h, washed with distilled water, incubated with 1% tannic acid (w/v) in 0.1 M cacodylate buffer for 1 h, washed with distilled water, then incubated with 1% osmium tetroxide in water (30 min). Samples were washed with distilled water and stained with 1% uranyl acetate (w/v) in water (1 h), dehydrated in graded ethanol then transferred to acetone prior to embedding into Agar100Hard resin.

### Atomic force microscopy

Fluorescence and brightfield imaging was used to guide Atomic force microscopy (AFM) imaging. 7 μm OCT-embedded unfixed sections were washed in PBS before incubation with an anti-Col4a2 antibody (H22), washed with PBS, and incubated with secondary antibody (15 min). AFM was performed using a JPK Nanowizard® 3 BioScience AFM (Bruker) in filtered dPBS ([-] calcium, [-] magnesium) at room temperature. Before imaging, cantilevers (PNP-DB from NanoWorld, 35° quadratic pyramidal tip, ∼0.48 N/m spring constant) were calibrated using a contact method on glass to determine sensitivity and thermal noise method to determine spring constant using the JPK SPM software. The tissue was scanned in Quantitative Imaging™ mode. Analysis was performed using JPK software by fitting the force curves with a Hertz model to obtain the Young’s modulus map alongside the contact point height image. From each animal 2 Bowman’s capsules were randomly selected and imaged, and the average used for final analysis. Image analysis was carried out by overlaying a grid on each image and measuring BM stiffness at each cross point in contact with Bowman’s capsule (30–40 random measurements per image). Due to technical reasons leading to daily variation in absolute values and inability to assess the entire cohort on a single day, relative values to wild type are provided in the graph for the cohort.

### Protein extraction

Brain vessel enriched fractions were prepared from frozen hemi-brains as described.^23^^,^^24^ Protein lysates of mesenteric vessels were prepared by mechanically removing surrounding fat. Protein extracts were obtained by tissue homogenization (TissueLyser, Qiagen) in RIPA buffer containing phosphatase (PhosSTOP, Roche) and protease inhibitors (Complete Mini, Roche).

**Western blotting** was performed using Mini PROTEAN® Electrophoresis system (Bio-Rad), proteins transferred onto nitrocellulose membranes (GE Healthcare). Membranes were stained using 0.5% ponceau S., de-stained in transfer buffer, and were blocked (5% milk, 5% or 3% BSA) before incubation with primary antibodies (Supplemental Table S2). Membranes were incubated with HRP-conjugated secondary antibodies (1 h, room temperature), developed using chemiluminescense (Millipore) and visualised (Chemidoc XRS+(Biorad), Amersham ImageQuant 800). Membranes were incubated with 30% H_2_O_2_ (20 min) or stripping buffer (Restore, ThermoFisher), washed in TBS before blocking and re-probing. Protein levels were corrected using Ponceau staining. Data was analysed using ImageJ.

### RT-PCR

Animal tissue was homogenised in TRIzol (Invitrogen) using a TissueLyser II (Qiagen), and RNA exacted per manufacturer’s protocol. RNA samples were DNase treated (TURBO DNA-free Kit, Invitrogen). cDNA synthesis was performed (high-capacity cDNA kit, ThermoFisher). qRT-PCR was carried out using Power Up SYBR green Mastermix (Invitrogen). Primer sequences in Supplemental Table S2. qRT-PCR data were analysed by 2ˆ-ΔΔCt method.

**Cell culture** Human brain microvascular endothelial cells (HBECs-5i, ATCC Cat# CRL-3245, RRID:CVCL_4D10) were cultured in DMEM:F12 (Gibco) supplemented with ECGS (Millipore), 10% FBS and 1% Pen/Strep on 0.1% Gelatin coated plasticware. For experiments cells were cultured in culturing media containing 0.25 mM L-Ascorbic acid (Sigma #A8960) for 72 h prior to analysis, as described.^9^ For cells cultured on collagen IV, plasticware was coated with gelatin and 50 μg/ml human collagen IV (#CC076, Merck), and cells were cultured for 72 h in culturing media containing 0.25 mM L-Ascorbic acid.

### Genome editing

Alt-R® CRISPR *crRNA* and tracrRNA (Integrated DNA Technologies) were mixed in equimolar ratio and annealed by heating (95 °C, 5 min) and slow-cooled to room temperature, prior to mixing with Cas9 protein (NEB). Guides (sequence: *COL4A1* G755R mutation: CGGCATTCCTGGCACACCCG; COL4A2 guides: CAGCCTGACGGTCCACGCTC, CTCATGCTTAAATGTGTCAC) were designed using RNA design web tool (IDT, Coralville, USA). Cells were transfected with Cas9 RNP using Lipofectamine CRISPRMAX Cas9 Transfection Reagent (Thermofisher) or TransIT-X2 (Mirus Bio) and phosphorothioate-modified ssODN (CAAAGGTTTGCCAGGTCTTCCCGGCATTCCTGGCACACCTAGGGAGAAGGGGAGCATTGGGGTACCAGGCGTTCCTGGAG) in presence of 20 μM SCR7 (Sigma) and 5 μM L-755,507 (Tocris BioScience). Cells were cultured for 48 h post-transfection and replated (density: 0.5 cells/well) prior to PCR and restriction enzyme-based screening for detecting edited cell lines. Sanger sequencing was used to confirm the mutation; Inference of CRISPR editing web tool (Synthego) to analyse HDR editing.

### HiPSC culture and differentiation

*COL4A1*^*G755R*^, COL4A2^G702D^ and isogenic hiPSC lines were generated as previously described.^15^ hiPSC lines were cultured in TeSR™-E8 media (STEMCELL Technologies) using Vitronectin XF (STEMCELL Technologies) as chemically defined xenofree cell culture matrix. All hiPSC lines were tested for mycoplasma by Mycoplasma Experience LTD. hiPSC-derived endothelial cells were differentiated using a previously reported protocol with minor modifications.^25^ Briefly, hiPSCs were seeded in TeSR™-E8 medium on vitronectin-coated 6-well plates (day 1) and after 24 h, E8 medium was replaced with BPEL (BSA poly (vinyl alcohol) essential lipids) medium supplemented with 8 μM CHIR. On day 3, medium was replaced with BPEL medium supplemented with VEGF-A (50 ng/ml; Peprotech) and SB431542 (10 μM; Tocris Bioscience) and refreshed on days 6–9. hiPSC-ECs were isolated on day 10 by sorting using MiniMACS separator and CD34 MicroBead kit (Miltenyi Biotec) and maintained in EC-SFM medium (ThermoFisher) + VEGF-A + bFGF2 (20 ng/ml, Peprotech).

### Ca^2+^ measurement

Cells were loaded with Fluo-4 Direct Calcium Assay Kit (Molecular Probes) and incubated for 60 min at 37 °C 5% CO_2_. Cells were imaged (>30 cells/microscopic field) using a Nikon DS-Fi3 camera on a Nikon Eclipse TS2R microscope. 100 μM acetylcholine was added for stimulation. Epifluorescence images were captured by time lapse acquisition mode in NIS-Elements BR, during a 1-min time-lapse. For basal Ca^2+^ levels in cells cultured on collagen IV, a 3 s timelapse was used. Image analysis was performed using CALIMA Calcium Imaging Tool.^26^ hiPSC-EC were preloaded with the calcium-sensitive fluorophore Fluo4AM (4 μM, Molecular Probes) in Krebs solution for 15 min at 37 °C. Cells were washed at room temperature and intracellular calcium flux was monitored as time series with acquisition rates of 1 frame every 1 min over 20 min using a Zeiss LSM 700 confocal microscope before and after addition of 100 μM acetylcholine. For experiments, five cells were randomly picked from a field of view, and the fluorescent trace was analysed using Fiji/ImageJ.

### Whole genome sequencing

Whole genome sequencing of two *Col4a2*^*+/em2Wtsi*^ mice was performed by Edinburgh Genomics. Reads were aligned to the mouse genome, version GRCm38, using BWA^27^ and alignments were recalibrated for base quality scores, realignment around insertion deletion sites, duplicate read removal and subsequent variant discovery using the Genome Analysis Toolkit (GATK)^28^ to produce Genomic Variant Call Format (gVCF) files. GATK was used to consolidate the gVCF files and perform joint variant calls on the single nucleotide variants (SNVs) and the insertions and deletions (INDELS) to produce a single file in VCF format. Variant calling was performed according to GATK Best Practices.^29^^,^^30^ Analysis and visualization were performed using custom R scripts utilizing the Gviz library.

### Patient cohort

We selected post-mortem paraffin-embedded human brain samples from the UK MRC Brain Bank (University of Edinburgh) and LINCHPIN study.^2^^,^^14^ Brain tissue was collected with informed consent from patients or their families. We randomly selected samples with a CSVD score of ≥2 based on neuro-imaging markers^31^ that did not contain any rare *COL4A1*/*COL4A2* variants, and selected all samples with sporadic ICH harboring rare *COL4A1*/*COL4A2* variants for which brain tissue was available (Supplemental Table S1). We used samples negative for ICH and CSVD as controls. For the patient cohort, with a sample size of 18 per group we have 80% power to detect a difference of at least 9% between groups in collagen IV staining as fraction of vessel wall area, assuming a within-group standard deviation of 9% (based on results from^32^) and Type I error rate of 5%.

### Analysis of human brain tissue

Collagen IV immunostaining and tissue was performed as previously published.^32^ Five vessels of arteriole appearance (size 40–150 μm least outer diameter) were randomly selected from each patient and imaged on Nikon Eclipse TS2R microscope. Lumen area fraction (AF) was calculated as AF = 100x (lumen area/total vessel area). Briefly, raw images were separated by colour channel and collagen IV labelled pixels were detected using an automatic threshold detection method (Examples in Supplemental Fig. S9). Collagen-IV positive area fraction (%) within each vessel wall was calculated as % = 100 × (collagen-IV positive vessel wall area/total vessel wall area) as previously described.^32^ Vessel wall area and thickness were measured using collagen IV staining delineating endothelial and brain parenchymal BMs. Image analysis was performed blind to clinical data using ImageJ.

**Statistical analysis** was performed using GraphPad Prism software. Shapiro–Wilk test was performed for normality testing. Welch’s, unpaired t-test or Mann–Whitney U test to compare means, Welch’s ANOVA with post hoc testing for multiple data points (post hoc testing include Dunnett’s test). Dose response curves were assessed using Area Under the Curve with post hoc testing. Kruskal Wallis test ANOVA with Bonferroni adjusted Mann–Whitney post hoc test was used when the data were not normally distributed. Fisher’s exact test was performed to compare presence/absence of phenotypes. p values less than 0.05 were deemed statistically significant and error bars in figures denote standard deviation. In the Figure legends 95% CI intervals and point estimate are provided for the difference between the means, except for Mann–Whitney calculations where closest confidence interval is provided.

### Role of funders

Funders had no role in study design, data collection, data analyses, interpretation, or writing of report.

## Results

### *Col4a1* glycine mutation increases vasodilation in CSVD with ICH

**Fig. 1:**
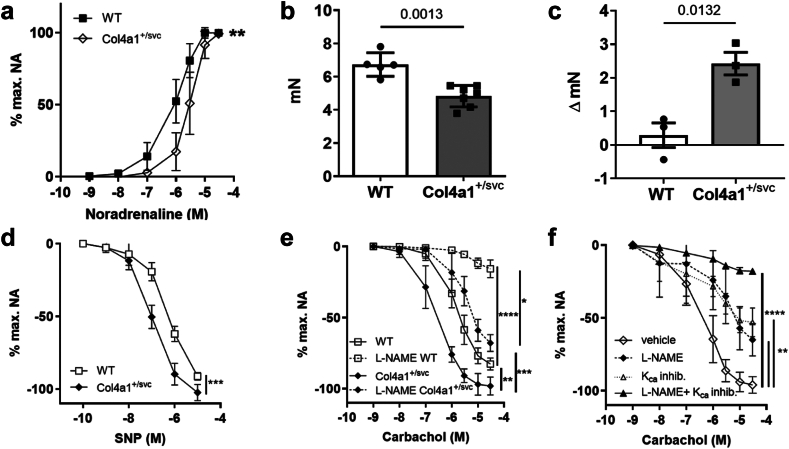
***Col4a1* glycine mutation causes vascular dysfunction.** (a) Reduced contraction to noradrenaline (NA) in *Col4a1*^*+/SVC*^ compared to wild type (WT) arteries (n = 5, area under the curve with followed by Welch’s t-test, point estimate −48.6 (95% CI: −69.63 to −27.57)). Graph of maximum constriction to noradrenaline is provided in Supplemental Fig. S1a. (b) 30% decreased maximal response to KCl in *Col4a1*^*+/SVC*^ (n = 5, Welch’s t-test, point estimate −1.91 (95% CI −2.83 to −0.99)). (c) Increased basal NO generation in *Col4a1*^*+/SVC*^ measured by increase in constriction of arteries to NA with pre-treatment of L-NAME compared to without L-NAME (n = 3, Welch’s t-test, point estimate 2.014 (95% CI 0.742–3.53)). (d) Increased endothelial cell independent vasodilation in *Col4a1*^*+/SVC*^ vessels shown by elevated dose response to SNP of vessels pre-constricted with NA (n = 5, area under the curve followed by Welch’s t-test, point estimate 68.7 (95% CI 50.08–87.32)). (e) Greater endothelial dependent relaxation in *Col4a1*^+/*SVC*^ vessels indicated by increased vasodilation to carbachol. Pre-treatment with NOS inhibitor L-NAME shows contribution of eNOS mediated vasodilation is reduced in mutant arteries (n = 4–7; area under the curve and Welch’s ANOVA with Dunnett’s T3 multiple comparison test; WT versus L-NAME WT p < 0.0001 point estimate 101.435 (95% CI 86.67–116.2), WT versus *Col4a1*^*+/SVC*^ p = 0.0055, point estimate −88.42 (95% CI −129.0 to −47.85), L-NAME WT versus L-NAME *Col4a1*^*+/SVC*^ p = 0.0293, point estimate −62.84 (95% CI −110.6 to −15.09), *Col4a1*^*+/SVC*^ versus L-NAME *Col4a1*^*+/SVC*^ p = 0.0007 point estimate 127 (95% CI 86.78–167.2). (f) *Col4a1*^*+/SVC*^ vessels pre-treated with K_Ca_ inhibitors (K_Ca_ inhib) apamin and TRAM-34 reduce endothelium dependent relaxation by ∼50%. Endothelium dependent vasorelaxation in *Col4a1*^*+/SVC*^ vessels pre-treated with apamin, TRAM-34 and L-NAME was similar to WT vessels pre-treated with L-NAME, indicating increased EDH vasodilation in mutant arteries (n = 4–7, area under the curve and Welch’s ANOVA with Dunnett’s T3 multiple comparison test; SVC vehicle versus SVC LNAME p = 0.0033, point estimate 88.99 (95% CI 38.49–139.5), SVC vehicle versus SVC LNAME + K_Ca_ inh p < 0.0001, point estimate 159.6 (95% CI 130.1–189.1), SVC vehicle versus SVC + K_Ca_ inh p = 0.0016, point estimate 82.815 (95% CI 41.53–124.1)) ∗p < 0.05, ∗∗p < 0.01, ∗∗∗p < 0.001, ∗∗∗∗p < 0.0001.

We set out to investigate CSVD mechanisms by assessing vascular function in small mesenteric arteries of a mouse model of Gould syndrome, *Col4a1*^*+/SVC*^ that carries a heterozygous missense mutation in *Col4a1* (G1064D)^11^^,^^20^ which substitutes a glycine residue for aspartic acid in the collagen domain of α1α1α2(IV). This mutation is representative of ∼60% of mutations in Gould Syndrome.^33^ We analysed 3-month-old *Col4a1*^*+/SVC*^ mice, that have CSVD with ICH as this age,^11^^,^^34^ which showed reduced sensitivity and pressor responses to noradrenaline (Fig. 1a and b, Supplemental Fig. S1a). We ascribe these changes due to the vasodilatory effect of increased basal NO generation because *Col4a1*^*+/SVC*^ vessels showed enhanced vasoconstriction induced by the NOS inhibitor L-NAME (Fig. 1c, Supplemental Fig. S1b). These changes were compounded by increased responsiveness to NO shown by responses to the NO-donor sodium nitroprusside (SNP) (Fig. 1d). To unpick the impact of *Col4a1* glycine mutations on vasodilation, we investigated the NO-cGMP, prostacyclin and EDH-mediated pathways. Surprisingly, dose response curves to carbachol revealed an increased vasodilation in *Col4a1*^*+/SVC*^ mice (Fig. 1e, Supplemental Fig. S1c) with a reduced relative contribution of the NO-cGMP pathway, revealed by inhibiting NO synthesis with L-NAME (Fig. 1e), despite the increased vessel responses to NO and increased basal NO levels. These data indicate increased activation of prostacyclin- and/or EDH-mediated vasodilation but no difference was detected in prostacyclin-mediated vasodilation (Supplemental Fig. S1d). However, the similar extent of vasorelaxation in *Col4a1*^*+/SVC*^ vessels pre-treated with apamin, TRAM-34 (which inhibit small and intermediate K_Ca_ [SK_Ca_, IK_Ca_], respectively) and L-NAME compared to WT vessels pre-treated with L-NAME alone, established increased EDH vasodilation in CSVD (Fig. 1f). This vascular dysfunction and CSVD occurs independent of hypertension (Supplemental Fig. S1g), which is a leading risk factor for CSVD.^3^ Thus, *Col4a1* mutations cause dysregulated NO- and increased EDH vasodilation via the small and intermediate K_Ca_ channels, revealing a new mechanism for small vessel disease.

### Reduced collagen IV levels increase the risk for adult-onset microhemorrhage

The mechanisms of missense mutations, rare coding variants and common-risk alleles of *COL4A1/COL4A2* in CSVD with ICH are poorly understood. Both BM defects and ER stress caused by missense mutations occur in mice and patients with *COL4A1*/*COL4A2* mutations^5^^,^^9^^,^^11^^,^^35^ but their relative contribution to mechanisms of COL4-associated CSVD is unknown and an important gap in our knowledge.

**Fig. 2:**
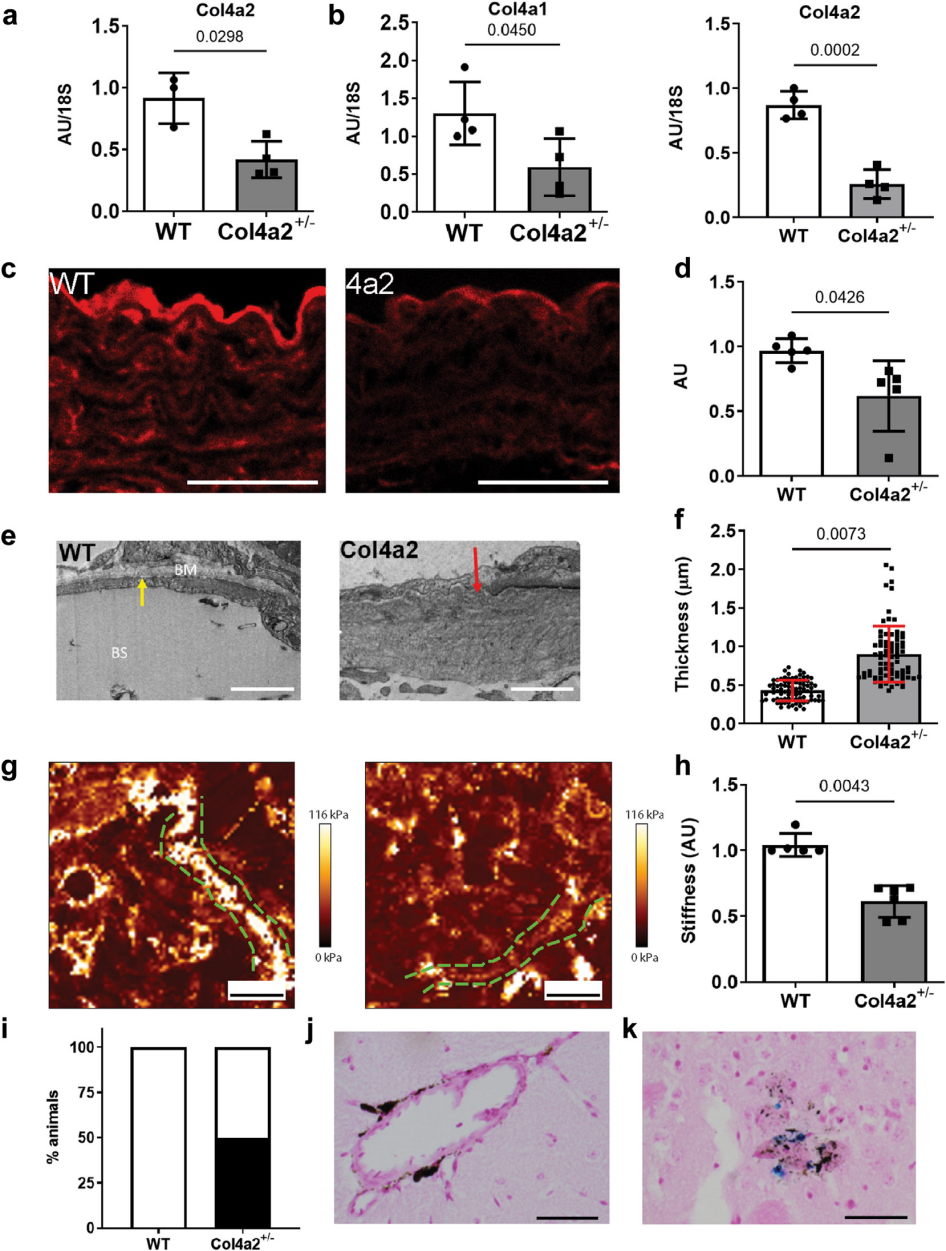
**Basement membrane defects in *Col4a2***^***+/em2Wtsi***^**mice.** (a) Reduced *Col4a2* mRNA levels in cerebrovasculature of 6-month-old *Col4a2*^*+/em2Wtsi*^ mice (Col4a2^+/−^) (n = 3–4, Welch’s t-test, point estimate −0.4963 (95% CI −0.9081 to −0.08450)). (b) Lower *Col4a1* and *Col4a2* mRNA levels in mesenteric arteries of 6 month old *Col4a2*^*+/em2Wtsi*^ mice (n = 3–4, Welch’s t-test, *Col4a1* point estimate −0.611 (95% CI −0.8004 to −0.4220); *Col4a2* point estimate −0.71 (95% CI −1.398 to −0.02204)). (c) Immunostaining (red) against α2(IV) in aorta of wild type (WT) and Col4a2^+/em2Wtsi^ (4a2) mice. Scale bar 50 μm. (d) Quantification of staining in (c) (n = 5, Welch’s t-test, point estimate −0.35 (95% CI −0.6827 to −0.01735)). (e) Multiple strand formation in BM of kidney Bowman’s capsule (red arrow, see also Supplemental Fig. S4 for vascular and tubular BM in kidney) Scale bar 2 μm. (f) Increased thickness of Bowmans Capsule BM in *Col4a2*^*+/em2Wtsi*^ (data points are individual measures from n = 3 mice, Welch’s t-test applied on n = 3, point estimate 0.4708 (95% CI 0.3873–0.5543)) (g) Atomic force microscopy images displaying stiffness of the BM of Bowman’s capsule (green dotted line) in WT and *Col4a2*^*+/em2Wtsi*^. Scale bar 10 μm. See also Supplemental Fig. S3 for exemplar AFM images of height and stiffness (same image provided), and immunostaining used to guide AFM. (h). Reduced BM stiffness in *Col4a2*^*+/em2Wtsi*^ (n = 5–6, Mann–Whitney U Test, point estimate: −0.3389 (96.97% CI −0.5588 to −0.2884)). (i) Presence of positive hemosiderin staining in 50% of *Col4a2*^*+/em2Wtsi*^ (n = 6, Fisher’s exact test p = 0.18). (j) Pigment deposit in *Col4a2*^*+/em2Wtsi*^ Scale bar 50 μm. (k) Hemosiderin positive stain (blue) in *Col4a2*^*+/em2Wtsi*^. Scale bar 50 μm.

To shed light on this we obtained *Col4a2* heterozygous knock-out mice (*Col4a2*^*+/em2Wtsi*^) that harbor a deletion of exon 18 by CRISPR.^36^ We confirmed the *Col4a2* deletion and absence of other coding variants (Supplemental Fig. S2a and b). Heterozygous *Col4a2*^*+/em2Wtsi*^ mice displayed normal Mendelian ratios at weaning and body weight, and had reduced *Col4a2* mRNA and protein levels (Fig. 2a–d, Supplemental Fig. S2c–g). *Col4a1* mRNA levels did not always correlate with *Col4a2* levels (Fig. 2b; Supplemental Fig. S2h–j), indicating, at least to some extent, independent regulation despite their shared promoter.^5^^,^^37^ No ER stress was detected in the cerebrovasculature or kidney of *Col4a2*^*+/em2Wtsi*^ (Supplemental Fig. S2k–o), establishing any phenotypes are due to reduced collagen IV levels, which cause BM thickening and splitting (Fig. 2e and f; Supplemental Fig. S3a–d), with reduced BM stiffness revealed by atomic force microscopy (Fig. 2g and h, Supplemental Fig. S3e). The atomic force microscopy was performed on the BM of Bowmans Capsule because it contains α1α1α2(IV) similar to the vascular BM, and the proximity of the vascular lumen to the BM prevented measurements. *Col4a2*^*+/em2Wtsi*^ mice can therefore be used to determine how BM defects due to reduced collagen IV levels cause disease.

Mice with *Col4a1* missense mutations develop anterior segment dysgenesis, renal defects and cerebrovascular disease by 3 months of age.^19^^,^^20^^,^^38^^,^^39^ In contrast, 14-month-old *Col4a2*^*+/em2Wtsi*^ mice showed no overt signs of anterior segment dysgenesis (Supplemental Fig. S4m). In the kidney, capillary tuft retraction and thickening of Bowmans Capsule with mild polyuria and polydipsia were observed but no hydronephrosis, medullary atrophy or fibrosis (Supplemental Fig. S4a–l). This indicates a later onset and milder disease compared to *Col4a1* missense mutations^11^^,^^19^^,^^20^ in line with mice with a *Col4a2* missense mutation.^40^ Half (3/6) of 14-month-old *Col4a2*^*+/em2Wtsi*^ mice develop micro-ICH, a manifestation of CSVD, shown by hemosiderin staining (Fig, 2i-k, Supplemental Fig. S4n). A brown-yellow pigment deposit was observed in every *Col4a2*^*+/em2Wtsi*^ mouse (6/6, p = 0.0022, Fisher’s exact test) in the vascular wall or co-occurring with hemosiderin staining (Fig. 2j and k), suggesting this reflects lipofuscin or ceroid and/or intact red blood cells.^41^ Thus, reduced collagen IV levels increase the risk of late-onset ICH and kidney disease, and suggest common risk alleles in *COL4A2* for sporadic adult-onset CSVD with ICH act by reducing collagen IV levels.

### BM defects due to reduced collagen IV levels underlie vascular dysfunction

**Fig. 3:**
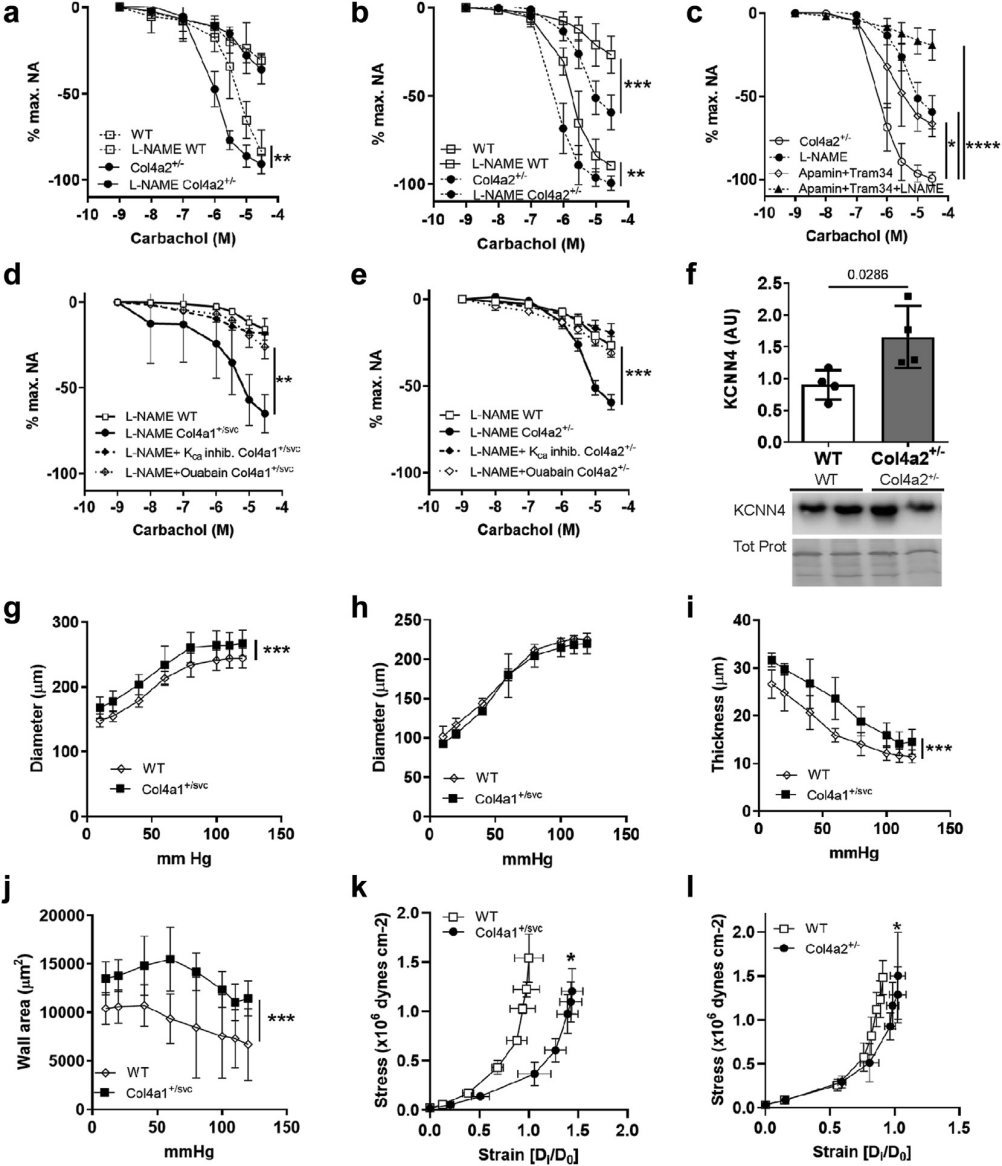
**Vascular defects due to reduced collagen IV levels.** (a) Dose response curve to carbachol in presence and absence of NOS inhibitor L-NAME in mesenteric arteries of 3-month-old wild type (WT) and *Col4a2*^*+/em2Wtsi*^ (Col4a2^+/−^) (WT versus *Col4a2*^*+/em2Wtsi*^ p = 0.0027, point estimate −50.35 (95% CI −77.88 to −22.82). Vasoconstriction and endothelium-independent vasodilation measured by SNP dose response curve is provided in Supplemental Fig. S5. (b) Dose response curve to carbachol in presence and absence of L-NAME (absence: WT, Col4a2^+/−^; presence: L-NAME) in 6-month-old mice reveals increased NO-dependent and -independent vasodilation in *Col4a2*^*+/em2Wtsi*^ (n = 4–5, WT versus Col4a2^+/em2Wtsi^ p = 0.0009, point estimate −46.3 (95% CI −68.51 to −24.09), WT L-NAME versus *Col4a2*^*+/em2Wtsi*^ L-NAME p = 0.0016, point estimate −31.69 (95% CI −47.43 to −15.95)). (c) Dose response curve to carbachol in presence of L-NAME (L-NAME), small and intermediate K_Ca_ channel blocker Apamin and TRAM-34, and L-NAME plus apamin and TRAM-34 shows EDH vasodilation in 6-month-old *Col4a2*^*+/em2Wtsi*^ (*Col4a2*^*+/em2Wtsi*^ versus *Col4a2*^*+/em2Wtsi*^ L-NAME p < 0.0001, point estimate 107.58 (95% CI 89.47–125.7); *Col4a2*^*+/em2Wtsi*^ versus *Col4a2*^*+/em2Wtsi*^ K_Ca_ Inh p = 0.0216, point estimate 72.86 (95% CI 19.03–126.7); *Col4a2*^*+/em2Wtsi*^ versus *Col4a2*^*+/em2Wtsi*^ L-NAME + K_Ca_ Inh, p < 0.0001 point estimate 140.8 (95% CI 120.3–161.3)). (d) Dose response curve to carbachol in presence of L-NAME, apamin and TRAM-34 (K_Ca_ inhibitor), and ouabain (inhibitor of Na^+^/K^+^ pump) in 3-month-old *Col4a1*^*+/SVC*^ reveals EDH is mediated by Na^+^/K^+^ pump. *Col4a1*^*+/SVC*^ L-NAME versus *Col4a1*^*+/SVC*^ ouabain, p = 0.0012 point estimate 0.001125 (95% CI 0.0007186−0.001532)) (e) Dose response curve to carbachol in presence of L-NAME, apamin and TRAM-34 (K_Ca_ inhibitor), and ouabain in 6-month-old *Col4a2*^*+/em2Wtsi*^ (*Col4a2*^*+/em2Wtsi*^ L-NAME versus *Col4a2*^*+/em2Wtsi*^ ouabain, p = 0.0005 point estimate 0.0006846 (95% CI 0.0004138–0.0009554)) (f) Increased protein levels in mesenteric arteries of intermediate K_Ca_ channel (KCNN4) in 6-month-old WT and *Col4a2*^*+/em2Wtsi*^ (Col4a2^+/−^). Tot. Prot: Ponceau stain of total protein (Mann–Whitney U test, n = 4, point estimate 0.6160 (97.14%CI 0.08814–1.689)). (g) Increased outer diameter of mesenteric arteries over range of pressures of 3-month-old *Col4a1*^*+/SVC*^ mice compared to wild type (WT) (p = 0.0003, point estimate 2553 (95% CI 2261–2845)). (h) Inner diameter of mesenteric arteries of 3-month-old wild type (WT) and *Col4a1*^*+/SVC*^ mice. (i) Elevated arterial wall thickness of 3-month-old *Col4a1*^*+/SVC*^ mice compared to wild type (WT) (p = 0.0003, point estimate 567 (95% CI 383.1–750.9)) (j) Increased cross sectional wall area of 3-month-old wild type *Col4a1*^*+/SVC*^ mice (p = 0.0003, point estimate 517,036 (95% CI 358,122–675,950)) (k) Stress–strain curve shows reduced vascular stiffness in 3-month-old *Col4a1*^*+/SVC*^ (p = 0.0263, point estimate −0.0608 (95% CI −0.08236 to −0.03924)) (l) Reduced vascular stiffness in 6-month-old *Col4a2*^*+/em2Wtsi*^ (Col4a2^+/−^) (p = 0.0396, point estimate 0.05891 (95% CI 0.01022–0.1076)) (a–e n = 3–5, Area under curve and Welch’s ANOVA with Dunnett’s test for multiple comparison; g-l n = 5 Area under curve followed by Welch’s t-test n = 5). ∗p < 0.05 ∗∗p < 0.01 ∗∗∗p < 0.001 ∗∗∗∗p < 0.0001.

To investigate if *Col4a1* glycine mutations cause endothelial dysfunction via reduced collagen IV levels, we investigated vascular function in 3-month-old *Col4a2*^*+/em2Wtsi*^ mice. This showed increased NO-mediated vasodilation without altered vasoconstriction or endothelial independent vasodilation in the absence of hypertension (Fig. 3a, Supplemental Fig. S4o–r and 5a–d). The vascular dysfunction progresses with age as, similar to *Col4a1*^*+/SVC*^, 6-month-old *Col4a2*^*+/em2Wtsi*^ mice have increased EDH vasodilation mediated by the small and intermediate K_Ca_ channels and a reduced contribution of NO vasodilation (Fig. 3b and c, Supplemental Fig. S5e–h).

Transfer of endothelial cell hyperpolarization to VSMCs is necessary for EDH-mediated vasodilation. Opening of small and intermediate K_Ca_ channels (K_Ca2.3_ and K_Ca3.1_ respectively) on endothelial cells increases K^+^ concentration between endothelial cells and VSMCs. This activates inward rectifier K^+^ channels (Kir) and the ouabain sensitive Na^+^, K^+^ ATPase (Na^+^/K^+^ pump), causing VSMC hyperpolarization and relaxation.^42^^,^^43^ To determine if the Na^+^/K^+^ pump mediates the increased EDH, we pre-treated arteries of *Col4a1*^*+/SVC*^ and *Col4a2*^*+/em2Wtsi*^ with ouabain and assessed vasodilation in the presence of L-NAME (Fig. 3d and e). The similar reduction in relaxation as with L-NAME combined with K_Ca_ channel inhibitors confirms the EDH is mediated by the Na^+^/K^+^ pump, and not via Kir. This is accompanied by increased levels of the intermediate K_Ca3.1_ channel (Fig. 3f, Supplemental Fig. S6d). We did not detect differences in the mRNA levels of small K_Ca_ channels (K_Ca2.1_, K_Ca2.2_, K_Ca2.3_), *eNOS*, and *Cav1* (caveolin) that can also affect EDH^44^ (Supplemental Fig. S6). This establishes that collagen IV and the BM are regulators of NO and EDH-vasodilation, and that *Col4a1/Col4a2* mutations cause vascular dysfunction in CSVD by reduced collagen IV levels.

### Collagen IV variants affect vascular biomechanics in CSVD

Vascular dysfunction can lead to vascular remodeling that occurs in CSVD with vascular wall thickening and increased stiffness. Vascular remodeling is largely modulated by mechanical forces generated by the blood flow and the tissue acting on the endothelium and VSMCs.^45^ Thus, to provide mechanistic insight into CSVD we determined the impact of collagen IV mutations on vascular structure, remodeling and biomechanics. Pressure myography revealed enlarged outer diameter and wall thickness in 3-month-old *Col4a1*^*+/SVC*^ mice (Fig. 3g and h) with increased wall/lumen ratio and cross-sectional wall area (Fig. 3i and j), indicating wall thickening and hypertrophic remodeling. Vascular remodeling and biomechanical properties go hand in hand, in which the ECM and VSMC play a key role^45^ and increased stiffness is associated with CSVD.^46^ Thus, we investigated biomechanical properties by determining the strain (vessel distension under a given stress) and stress on the vascular wall. This revealed a significant reduction in vascular stiffness in 3 month old *Col4a1*^*+/SVC*^ (Fig. 3k), with a significant increase in strain (∼30% at maximum pressure) showing more deformation of vessels in *Col4a1*^*+/SVC*^, but no overall difference in wall stress (Supplemental Fig. S1e and f). Six month old *Col4a2*^*+/em2Wtsi*^ mice showed that reduced collagen IV levels reduce vascular stiffness (Fig. 3l, Supplemental Fig. S5m and n), but we failed to detect altered wall thickness and diameter or hypertrophic remodeling (Supplemental Fig. S5j–l). This could reflect difference in disease stage whereby in *Col4a2*^*+/em2Wtsi*^ the wall thickening has not yet developed,^47^ and/or pleiotropy in mechanisms of *Col4a1* glycine mutations. These data establish collagen IV plays a role in maintaining vascular stiffness and that CSVD involves biomechanical vessel defects with reduced vascular stiffness via lower collagen IV levels and hypertrophic remodeling leading to wall thickening.

### Increased Ca^2+^ signaling in endothelial cells

**Fig. 4:**
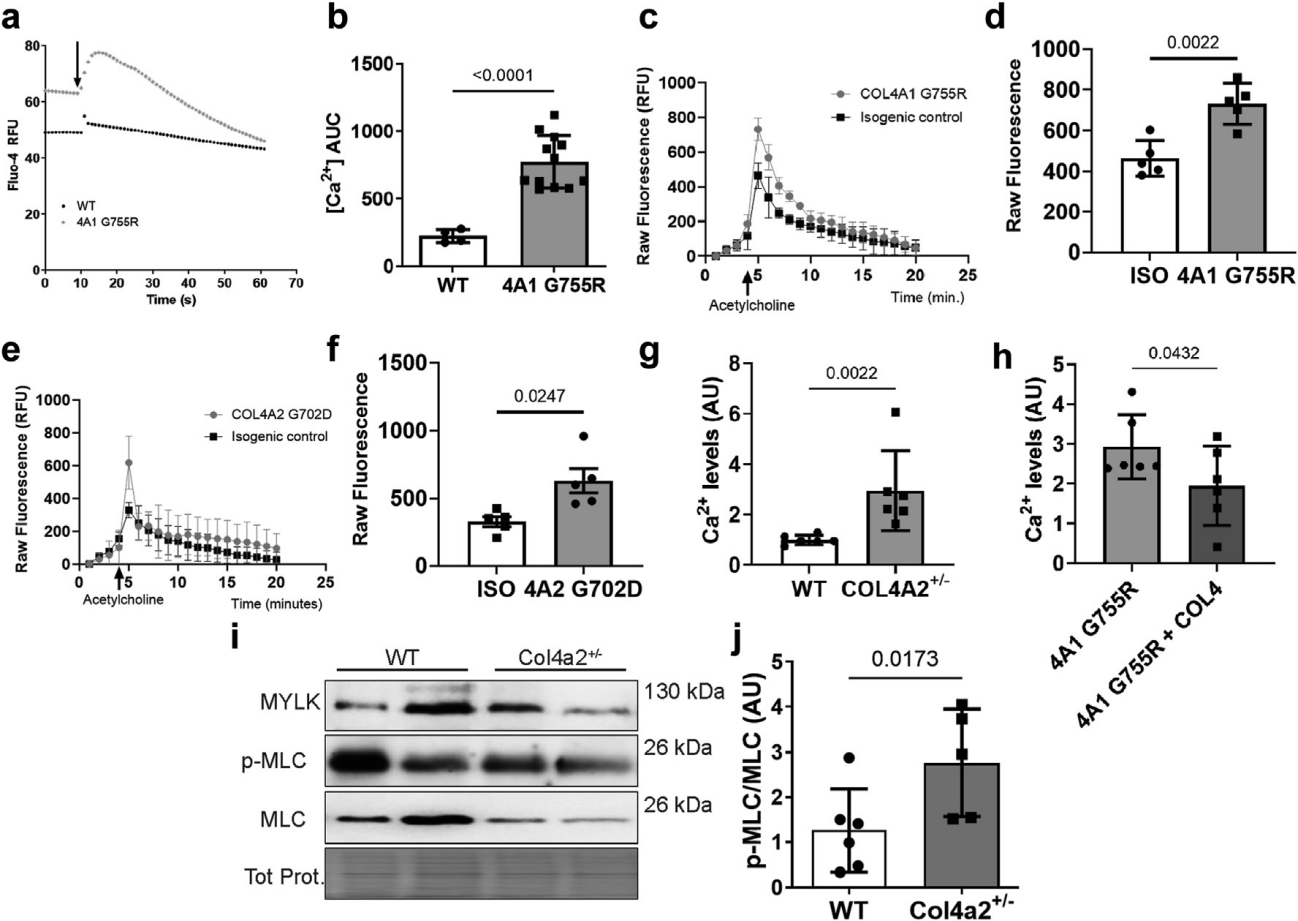
***COL4A1/COL4A2* variants increased Ca**^**2+**^**levels in brain endothelial cells.** (a) Increased basal intracellular calcium levels and after acetylcholine stimulation in human brain microvascular endothelial cell line (HBEC) harboring a *COL4A1* mutation measured using Fluo-4 Ca^2+^ tracer (RFU:relative fluorescent units). WT: wild type; 4A1 G755R: COL4A1^+/G755R^. (b) Quantification of intracellular Ca^2+^ concentration in *COL4A1* mutant cells (area under the curve analysis of graphs in (a) (AUC) with Welch’s t-test; wild type n = 4, Col4a1 G755R n = 12, point estimate 550.2 (95% CI 419.0–681.4)). (c) Trace of intracellular Ca^2+^ levels in human iPSC-derived brain endothelial cells showing increased response to acetylcholine in cells carrying *COL4A1* G755R mutation compared to isogenic control (iso). (d) Average peak value of (c) show increased cytoplasmic Ca^2+^ levels in mutant hIPSC-derived brain endothelial cells (Welch’s t-test, n = 5, point estimate 267.4 (95% CI 129.3–405.5)). (e) Trace of intracellular Ca^2+^ levels in *COL4A2* mutant (G702D) and isogenic control human iPSC-derived brain endothelial cells (n = 5). (f) Average peak value of (e) show cytoplasmic Ca^2+^ levels in *COL4A2* G702D mutant and isogenic control hIPSC-derived brain endothelial cells (Welch’s t-test, n = 5, point estimate 300.08 (95% CI 55.76–544.4)) (g) Basal intracellular Ca^2+^ levels in *COL4A2*^*+/−*^ HBEC cell line and WT (n = 6, Mann–Whitney U test, point estimate 1.528 (95.89% CI 0.8466–4.757)). (h) Effects of extracellular collagen IV levels via coating wells with COL4 on basal intracellular Ca^2+^ levels in *COL4A1*^*WT/G755R*^ HBEC cells (uncoated: 4A1 G755R; collagen IV coated: 4A1 G755R + COL4; n = 6, Paired t-test, point estimate −0.981 (95% CI −1.918 to −0.04396)). (i) Western blot against myosin light chain kinase (MLK), total and phosphorylated myosin light chain (MLC, p-MLC) in mesentery of 6-month-old *Col4a2*^+/−^ mice. Tot Prot: Ponceau stain of total protein (j) Increased ratio of phosphorylated: total MLC in mesentery of *Col4a2*^+/−^ mice. p = 0.0173 Mann–Whitney U test, point estimate 1.757 (96.97% CI 0.03886–3.258)).

Endothelial calcium signaling is critical for NO- and EDH-vasodilation whereby activation of eNOS and K_Ca_ channels depends on an influx of Ca^2+^. Altered endothelial calcium levels could therefore be a mechanism by which *Col4a1* mutations activate these pathways. We introduced a heterozygous *COL4A1* G755R mutation, which causes Gould syndrome,^48^ into a human microvascular brain endothelial cell line (HBEC) via CRISPR (Supplemental Fig. S7a–c) to measure basal Ca^2+^ levels and in response to acetylcholine. This revealed a significant increase in basal cytoplasmic calcium ion levels and in response to acetycholine (Fig. 4a and b). We also confirmed this using human iPSC-derived brain endothelial cells (Fig. 4c–f) that were established from patients with Gould syndrome due to a *COL4A1*^*G755R*^ and *COL4A2*^*G702D*^ mutation.^9^^,^^15^^,^^49^ To probe the link between extracellular collagen IV levels and basal cytoplasmic calcium levels, we used CRISPR to create HBECs that harbor a heterozygous deletion in exon 18 of *COL4A2* (Supplemental Fig. S7), equivalent to *Col4a2*^*+/em2Wtsi*^ mice, and found these cells have increased basal calcium levels (Fig. 4g). Furthermore, culturing *COL4A1*^+/G755R^ HBECs on collagen IV coated wells, as a model of increasing extracellular collagen IV levels, ameliorated the calcium levels (Fig. 4h). These data establish that collagen IV mutations lead to elevated intracellular calcium levels in endothelial cells and that collagen IV levels represent a therapeutic target.

To determine if collagen IV mutations induce a more “synthetic” phenotype in endothelial cells and lead to increased ECM production, RT-PCR was performed on the *COL4A1*^+/G755R^ cell line (Supplemental Fig. S7d), and we analysed the transcriptomic dataset of the IPSC-derived models.^15^ This revealed no evidence of a more synthetic phenotype by these cells.

Myosin light chain phosphorylation (MLC) is Ca2^+^ dependent, and increased ratio of phospho-MLC versus total MLC (Fig. 4i and j, Supplemental Fig. S8) in the mesentery of 6-month-old *Col4a2*^*+/em2Wtsi*^ mice provides *in vivo* support for increased Ca^2+^ signaling^50^ caused by reduced collagen IV levels. The unaltered levels of vimentin, αSMA and total MLC indicate there is no increase in the number of VSMCs and provide evidence for a contractile state of the VSMCs in the face of increased vasodilation (Supplemental Fig. S8). This indicates functional VSMC defects are part of collagen IV-associated CSVD.

### Genotype-dependent mechanisms of *COL4A1/COL4A2* variants in sporadic CSVD with ICH

**Fig. 5:**
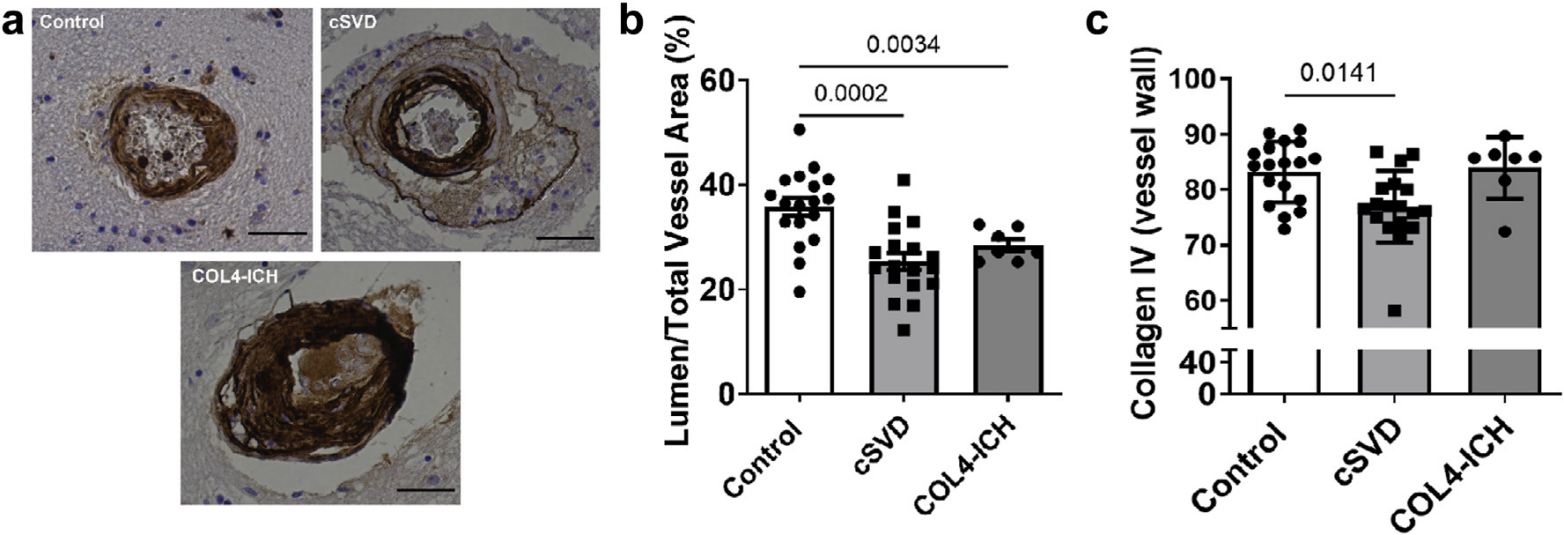
**Collagen IV levels and wall thickness in human sporadic CSVD.** (a) Immunostaining against collagen IV on brain tissue from non-ICH non-CSVD controls (n = 18), sporadic CSVD with ICH without rare coding *COL4A1/2* variants (CSVD) (n = 18), and sporadic CSVD with ICH harboring rare *COL4A1/2* variants (COL4-ICH) (n = 7) Size Bar 50 μM. (b) Vessel wall thickening in sporadic CSVD without rare COL4 variants (CSVD) and sporadic CSVD with ICH harboring rare COL4 variants (COL4-ICH). Vessel wall thickness: ratio of vascular lumen over total vessel area defined by parenchymal basement membrane stained with collagen IV (see Supplemental Fig. S9). Welch’s ANOVA with Dunnet’s test. Control versus cSVD point estimate 10.415 (95% CI 4.901–15.93), control versus COL4-ICH point estimate 7.313 95% CI (2.396–12.23). (c) Quantification of collagen IV staining as fraction of vessel wall area shows reduced levels in sporadic CSVD without rare COL4 variants sporadic (CSVD). Sporadic ICH with rare COL4 variants (COL4-ICH). Kruskal Wallis test ANOVA with Bonferroni adjusted Mann–Whitney post hoc test control versus cSVD p = 0.0141, point estimate 7.89% (95.35% CI: 0.76%–11.43%).

Common non-coding variants in *COL4A2* are genetic risk alleles for CSVD and ICH^7^^,^^8^ with the risk alleles being present in ∼65% of the population.^7^ Our previous sequence analysis of *COL4A1/COL4A2* in patients with sporadic CSVD with ICH in the LINCHPIN (Lothian INtraCerebral Haemorrhage Pathology Imaging and Neurological outcomes study) cohort also identified rare coding *COL4A1/COL4A2* variants in these patients.^6^ To shed light on the role of common non-coding *COL4A2* variants we interrogated brain tissue of randomly selected patients without rare coding *COL4A1*/*COL4A2* variants, and controls without ICH or cSVD (Supplemental Table S1). These patients had no other explanation for their ICH (e.g. macrovascular cause, tumour etc), and CSVD was therefore the only pathological substrate. Immunostaining of the basal ganglia area revealed wall thickening with lower collagen IV levels in small cerebral arteries (Fig. 5a–c), supporting our *Col4a2*^*+/em2Wtsi*^ mice data and that common non-coding *COL4A2* risk variants act by reducing collagen IV levels. Analysis of brain tissue of patients with rare coding *COL4A1/COL4A2* variants (Supplemental Table S1) also showed increased wall thickness (Fig. 5a–c). No glycine mutations, equivalent to the *Col4a1*^+/SVC^ mutation and the major cause of early-onset CSVD in Gould-syndrome, were identified in sporadic late-onset CSVD with ICH.^6^ Intriguingly, cerebral vessels of patients with rare coding variants had apparent similar collagen IV levels to controls. This provides evidence that in late-onset sporadic CSVD coding variants do not affect collagen IV secretion, in contrast to *COL4A1/COL4A2* mutations in early-onset familial Gould syndrome.^5^ Rather, it supports they act by secreting variant collagen IV that then also causes vascular wall thickening. This supports CSVD with common non-coding and rare coding variants in *COL4A1/2* has genotype-dependent mechanisms whereby reduced extracellular collagen IV levels and secretion of mutant protein converge on vascular wall thickening, providing insight into a genotype-phenotype correlation of CSVD.

## Discussion

**Fig. 6:**
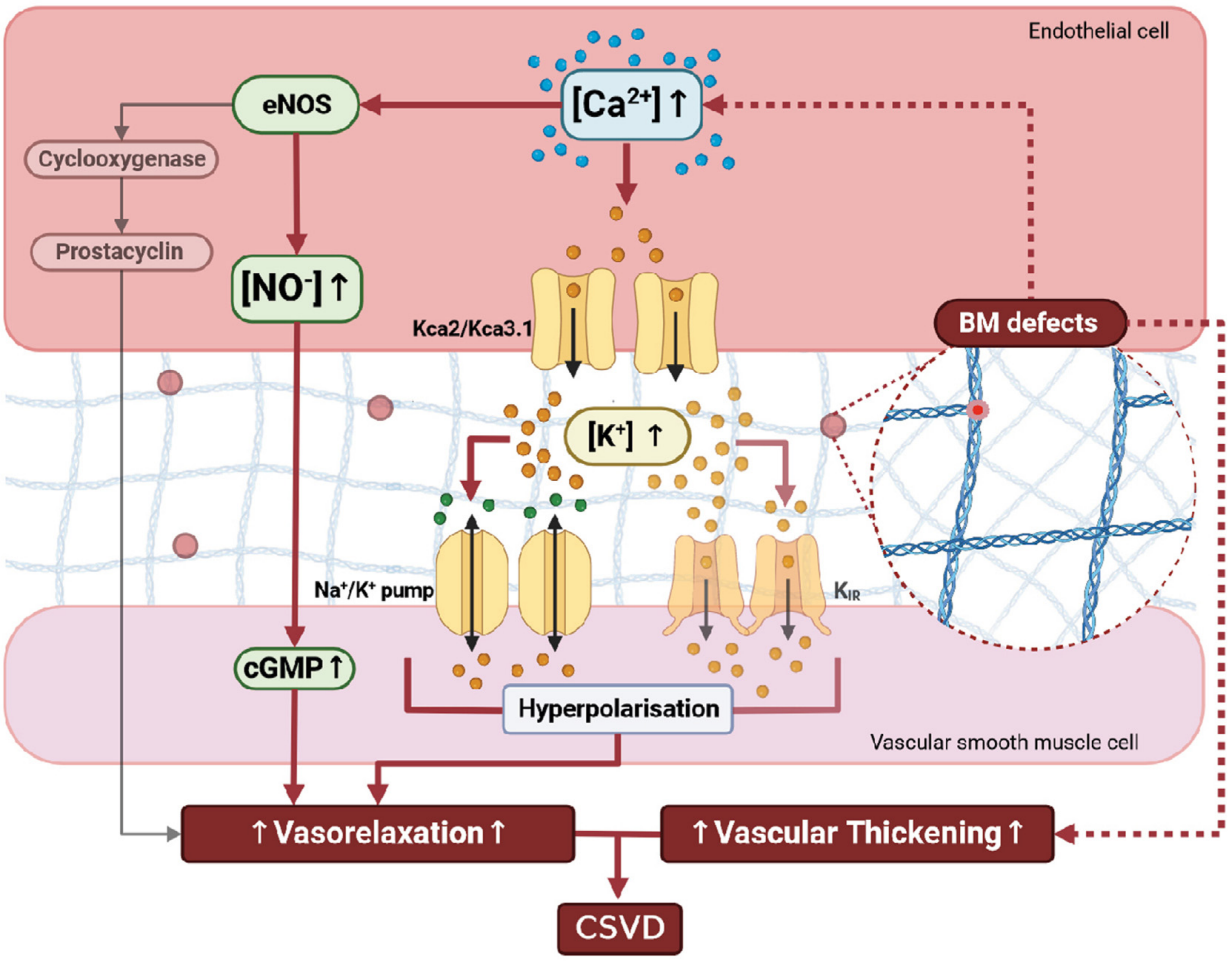
**Mechanisms of *COL4A1/COL4A2* variants in CSVD.** Diagram depicting overarching CSVD mechanism whereby basement membrane (BM) defects due to reduced collagen IV levels increase intracellular calcium levels leading to increased endothelial cell mediated vasodilation via EDH vasodilation mediated by K_Ca_ channels and Na/K pump. In addition, BM defects can also be due to mutant protein secretion, and both are coupled with vascular wall thickening in CSVD independent of hypertension.

Here, we established a mechanism for CSVD with ICH whereby collagen IV variants affect endothelial cell-mediated small vessel function and structure, uncovering a role for collagen IV in endothelium-dependent vasodilation. In mice, the CSVD occurred in the absence of hypertension, directly supporting the emerging concept that the mechanistic role of, at least some, cardiovascular risk factors on CSVD development may be more limited.^51^ The collagen IV variants cause increased EDH-mediated vasodilation via lower extracellular collagen IV levels with elevated calcium levels in endothelial cells and a contracted state of VSMCs. This leads to vascular hypertrophic remodeling with altered biomechanics with reduced stiffness (Fig. 6). The endothelial dysfunction occurs in early disease stages, 8 months before ICH in old age *Col4a2*^*+/em2Wtsi*^ mice, and thus confirms it is an initial driver of CSVD. Our data provide new insight into this poorly understood but critical aspect of CSVD, that to date had been most often linked to brain hypoperfusion and reduced vasodilation.^12^^,^^52^ It is tempting to suggest this increased dilation could progress to reduced perfusion in more-developed CSVD, providing insight into the complex relationship between blood flow and CSVD.^3^ However, as we investigated small peripheral vessels, it is now important to determine if these mechanisms are conserved in the cerebrovasculature.

Mechanistically, the enhanced vasodilation is due to BM defects with reduced collagen IV levels causing higher endothelial Ca^2+^ levels (Fig. 4a–f) that lead to opening of the K_Ca_ channels, associated with increased levels of the intermediate K channel, and activity of the Na^+^/K^+^ pump on VSMCs. The molecular link between matrix defects and enhanced Ca^2+^ levels remain unclear but could involve altered integrin signaling,^53^ which we have observed in patient cells harboring a *COL4A2* mutation.^54^ While our data exclude overt ER stress due to protein misfolding, a role for the ER in its capacity as calcium store herein cannot be excluded. The increased vasodilation is accompanied by vascular remodeling with increased thickness and a potential contractile state of VSMCs, rather than increased VSMC number, which could reflect a compensatory response. This is supported by reduced smooth muscle cell coverage in cerebral blood vessels upstream of segments with increased muscularization in ICH.^55^ This depletion and predicted vascular wall weakening has been proposed as a mechanism for the haemorrhage at this site by being unable to withstand higher intravascular pressure in the arteriole upstream of the hypermuscularisation in mice with *Col4a1* mutations.^55^

Missense mutations in *COL4A1/COL4A2* cause early-onset CSVD with ICH in COL4A1 (Gould) Syndrome,^9^^,^^33^^,^^56^ while rare missense variants and common non-coding variants have also emerged as risk factors.^6^^,^^7^ However, the upstream molecular insults of these different types of variants in CSVD, and by extension the basis and existence of a genotype-phenotype correlation, remained obscure. To date, as upstream molecular insults extracellular collagen IV deficiency, mutant protein secretion, and proteotoxic stress due to intracellular retention of misfolded protein have been proposed.^5^ In mice previous work by ourselves and others showed *Col4a1* missense mutations, including the *Col4a1*^*+/SVC*^ mutation, cause reduced extracellular collagen IV levels in multiple tissues,^10^^,^^11^^,^^19^^,^^35^ but the relative contribution of secreting mutant protein and reduced levels to the distinct phenotypes remains poorly understood, and is likely influenced by the mutation and/or tissue and cell type.^5^^,^^19^^,^^57^ Our data here establish that reduced extracellular collagen IV levels can cause endothelial dysfunction and late-onset CSVD with ICH in sporadic and genetic CSVD. This is supported by recent observational analysis indicating collagen IV levels negatively correlated with CSVD severity.^32^ Our data showing no difference in collagen IV levels in tissues from patients harboring rare putative pathogenic variants also provide evidence that secretion of mutant collagen IV is a second mechanism leading to vascular wall thickening in sporadic late-onset CSVD with ICH. This supports allele-specific mechanisms that converge on hypertrophic remodeling. The earlier age of onset and increased CSVD severity in *Col4a1*^*+/SVC*^ mice and Gould syndrome compared to sporadic CSVD could then be due to proteotoxic stress, as supported by our previous analysis of patients with a *COL4A2* mutation,^9^ and association of ICH severity with levels of collagen IV ER retention in mice,^35^ and/or even lower extracellular collagen IV levels. Combined, these data provide insight into genotype-phenotype relationships in CSVD. However, a limitation of our study is the sample size of our human cohort, which prevents us from accounting for confounders such as blood pressure or diabetes, which are known risk factors for CSVD.

In conclusion, our findings indicate a role for the BM and collagen IV in vascular function, and a mechanism for CSVD with vascular remodeling and enhanced EDH-vasodilation driven by lower collagen IV levels independent of hypertension. This significantly increases our mechanistic understanding of CSVD and suggests EDH and collagen IV levels as treatment targets.

## Contributors

SM, YYS, EB, GH, AA, AG, MC, ML, CG, YL, AK, CT performed the experiments, YT provided the collagen IV antibodies, KEK, MSS, WF, NB, TVA supervised the work. HM, MA, CV, CS and RA-SS provided patient data and tissue. Data analysis was performed by SM, YYS, EB, CT, AG, GH, MC, KEK, AA, MSS, AHH, JDM and TVA. SM, NB and TVA developed the project and concept. All authors assisted with writing the manuscript. Accession and Data verification was done by SM, CT and TVA. Funding was raised by CDA and TVA. All authors have read and approved the final manuscript.

## Data sharing statement

Data collected for the study and data from sample analyses will be made available upon reasonable request to the corresponding authors.

## Declaration of interests

CDA reports sponsored research support from the American Heart Association (18SFRN34250007 and 21SFRN812095) and Bayer AG, and consulting with ApoPharma, outside the scope of the current work. RA-SS reports grants outside the submitted work from BHF, Chief Scientist Office of the Scottish Government, and National Institutes of Health Research Health Technology Assessment programme paid to The University of Edinburgh, consultancy income paid to The University from Recursion Pharmaceuticals, and reimbursement for endpoint adjudication paid to The University of Edinburgh from NovoNordisk. TVA reports he serves on the grants committee of DEBRA UK and was honorary Treasurer for the British Society of Matrix Biology (2016–2022).

## Appendix ASupplementary data

Supplemental Figs. S1–S9 and Tables S1 and S2

Western Blots
