# Supplementary material for: Collagen IV deficiency causes hypertrophic remodeling and endothelium-dependent hyperpolarization in small vessel disease with intracerebral hemorrhage
Source: eBioMedicine. 2024 Aug 30;107:105315. doi: 10.1016/j.ebiom.2024.105315 (PMC11402910; doi:10.1016/j.ebiom.2024.105315)
Supplement: Supplemental Figs. S1–S9 and Tables S1 and S2 [file mmc1.docx]

**Collagen IV deficiency causes hypertrophic remodeling and endothelium-dependent hyperpolarization in small vessel disease with intracerebral hemorrhage.**

**Supplemental Figure 1**

**
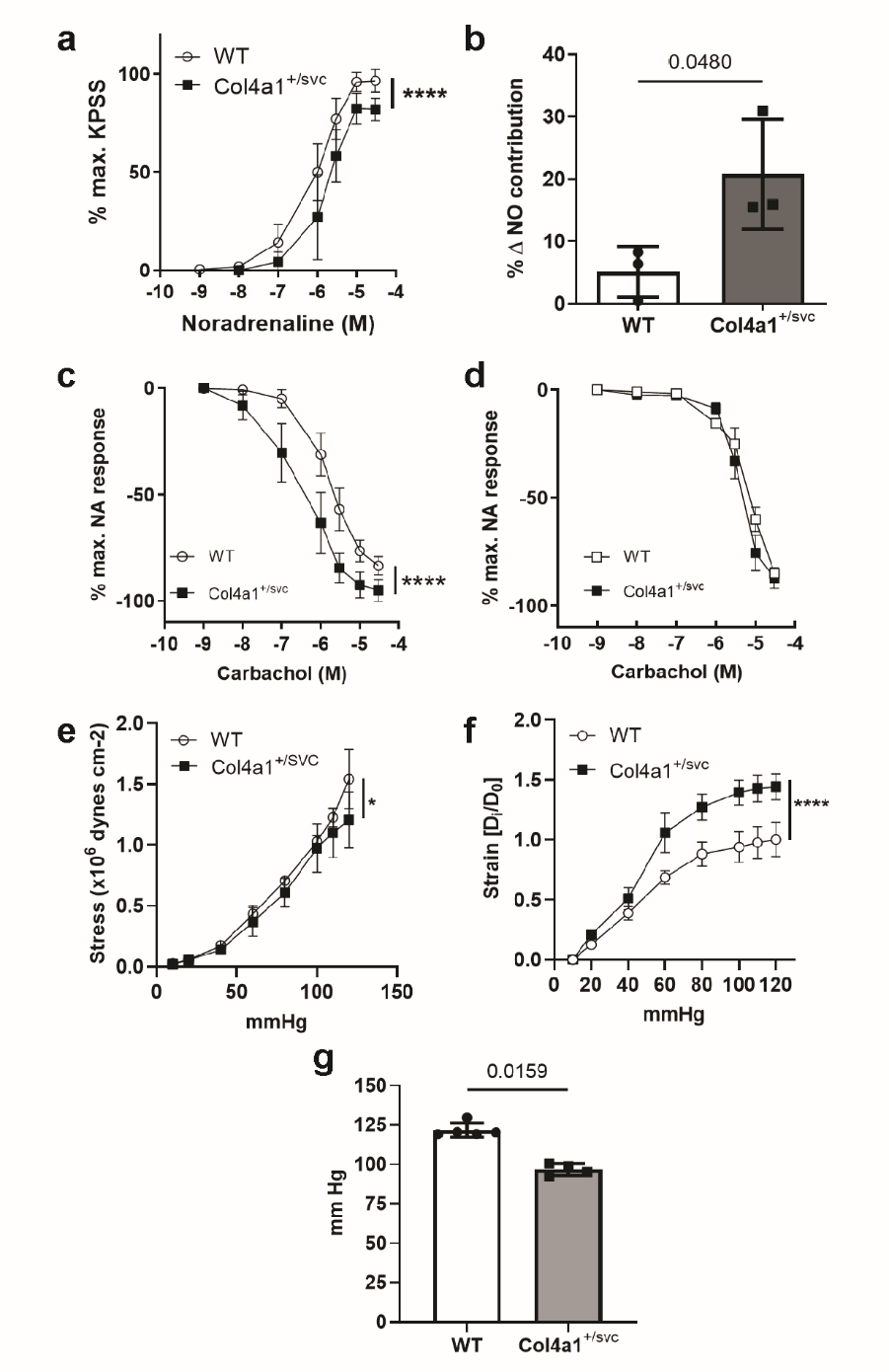
**

**Supplemental Figure 1 Dysfunction of small vessels in *Col4a1^+/SVC^* mice** (a) Decreased maximum response to noradrenaline in *Col4a1^+/SVC^* (n=5, area under the curve and Welch’s t-test p=0.0011, point estimate -0.000308 (95% CI -0.0004447 to -0.0001713) (b) Increased basal NO generation in mutant vessels determined indirectly by wire myography. Arteries were subjected to 10 μM noradrenaline with and without pre-treatment of L-NAME. Difference in level of constriction presented as % increase in vasoconstriction reflects the relative contribution of basal NO in WT and *Col4a1^+/SVC^*. (n=3, Mann-Whitney U test, point estimate 9.678 (90% CI 7.224 to 30.42)) (c) Greater endothelial dependent relaxation in *Col4a1^+/SVC^* vessels indicated by increased vasodilation to carbachol (Area under the curve with Welch’s t-test, n=4-7, point estimate 81.1 (95%CI 67.36 to 94.84)). (d) Assessment of prostacyclin mediated vasodilation measured by responses to carbachol in presence of the cyclooxygenase inhibitor indomethacin (10 μM). (n=4, Two-way ANOVA) (e) Stress of vascular wall over range of pressures in 3-month-old WT and *Col4a1*^+/SVC^ mice calculated from inner and outer diameter measures (n=5, Area under the curve and Welch’s t-test, p=0.0499, point estimate -7.81 (95% CI -15.61 to -0.006160 ). (f) Strain of vascular wall over range of pressures in 3-month-old WT and *Col4a1*^+/SVC^ mice, calculated from inner and outer diameter measures (n=5, area under the curve followed by Welch’s t-test, p<0.0001, point estimate 32.44 (95% CI 27.87 to 37.01)). (g) Blood pressure analysis using tail plethysmography (Mann-Whitney U test, n=4-6, point estimate -23.2 (96.83% CI -34.43 to -18.53)). (*p<0.05; **p<0.01; ***p<0.001)

**Supplemental Figure 2**

**
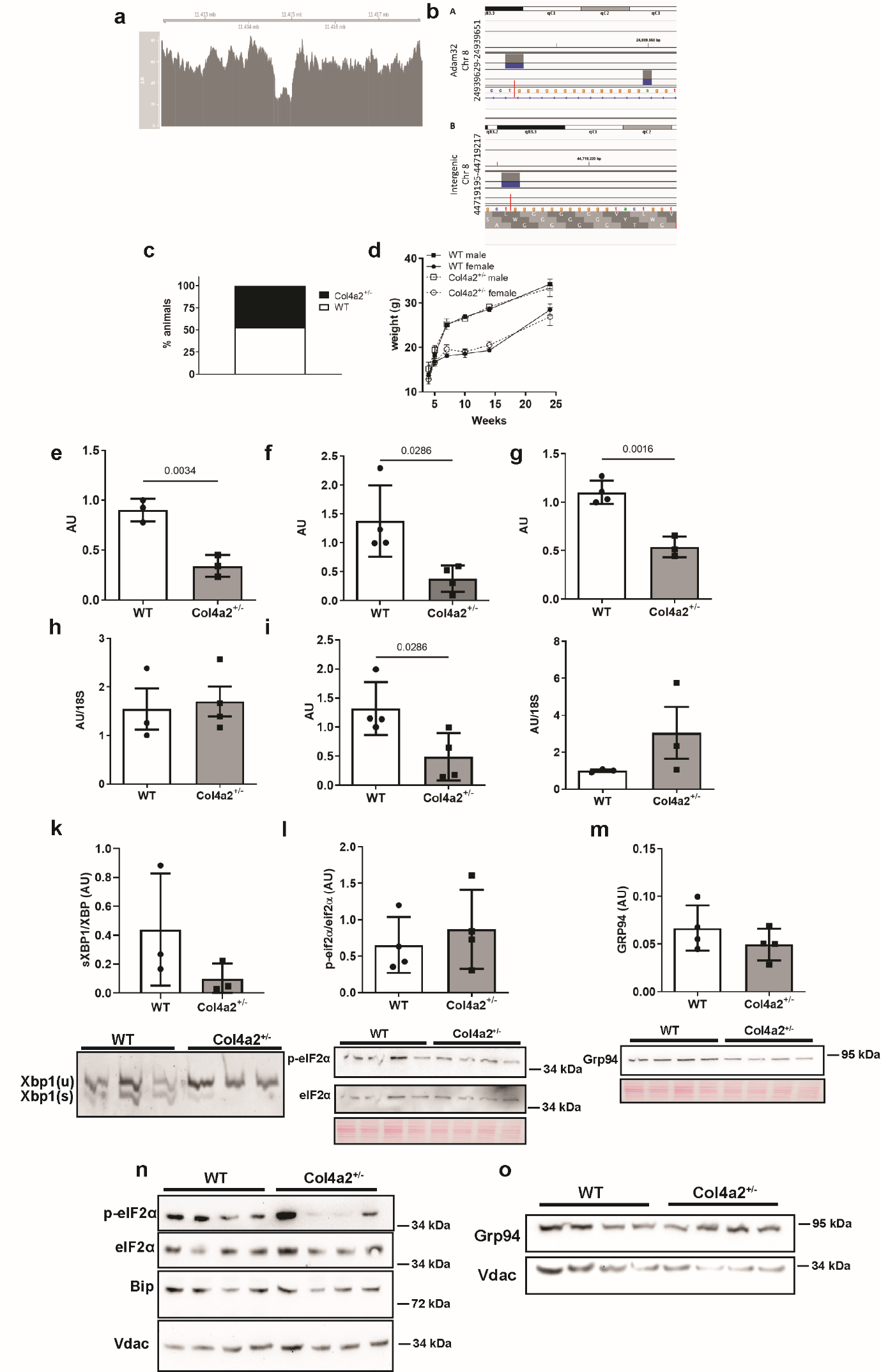
**

**Supplemental Figure 2** **Analysis of *Col4a2^+/em2Wtsi^* mice**. (a) reduced read depth indicates heterozygous deletion of exon 18 in *Col4a2^+/em2Wtsi^* mice. (b) Sequence analysis of predicted potential off-target mutations of sgRNAs (<https://wge.stemcell.sanger.ac.uk/>) used to generate *Col4a2^+/em2Wtsi^.*(1) Genome sequencing of 2 mice revealed 3 indels in the predicted 507 potential off-target sites. (A) In intron 1 of A Disintegrin And Metalloprotease (*ADAM*) 32, a 2 bp deletion was detected in one mouse and a 1 bp deletion 13 bp in the other mouse. (B) 2 bp deletion in an intergenic region on chromosome 8. All indels are near a guanine homopolymer sequence, suggesting these are sequencing read errors. Blue: deletions red: predicted cut site. (c) Mendelian ratios at weaning of mutant and wild type (WT) animals from WT x *Col4a2^+/^*^em2Wtsi^ (Col4a2^+/-^) crosses. (WT, n=86, *Col4a2^+/^*^em2Wtsi^ n=75) (d) Growth curve of wild type and *Col4a2^+/^*^em2Wtsi^  mice (n=6). (e-g) *Col4a2* mRNA levels in aorta (e; Welch’s t-test, n=3, point estimate -0.56 (95%CI -0.8119 to -0.3097)), kidney (f; Mann-Whitney U test, n=4, point estimate -0.700 (97.14% CI -2.192 to -0.3985)) and lung (g; Welch’s t-test n=3, point estimate -0.564 (95%CI -0.7897 to -0.3380)) of 6-month-old WT and *Col4a2^+/^*^em2Wtsi^ mice. (h-j) *Col4a1* mRNA levels in brain of 6-month-old WT and *Col4a2^+/^*^em2Wtsi^ mice (h; Welch’s t-test WT n=3, *Col4a2^+/^*^em2Wtsi^ n=4), kidney (i; Mann-Whitney U test, n=4, point estimate –0.728 (97.14%CI -1.851 to -0.006908)) and aorta (j; Welch’s t test, n=3). (AU arbitrary unit). (k) XBP1 and spliced XBP1 (Xbp1(s) mRNA levels in cerebrovasculature of 6-month-old wild type and *Col4a2^+/em2Wtsi^*. Ratio of spliced versus unspliced Xbp1 (Xbp1(u)) is provided in graph (Welch’s t-test, n=3). (l) Ratio of phosphorylated versus total eIF2α in cerebrovasculature of 6-month-old mice. Ponceau staining was used as protein loading control (Welch’s t-test n=4). (m) Grp94 protein levels in cerebrovasculature of 6-month-old *Col4a2^+/em2Wtsi^*. Ponceau staining was used as protein loading control (Welch’s t-test n=4). (n) Bip and ratio of phosphorylated versus total eIF2α protein levels in kidney of *Col4a2^+/em2Wtsi^*. VDAC was used as protein loading control. (o) Grp94 protein levels in kidney of *Col4a2^+/em2Wtsi^*. VDAC was used as protein loading control.

**Supplemental Figure 3**

**
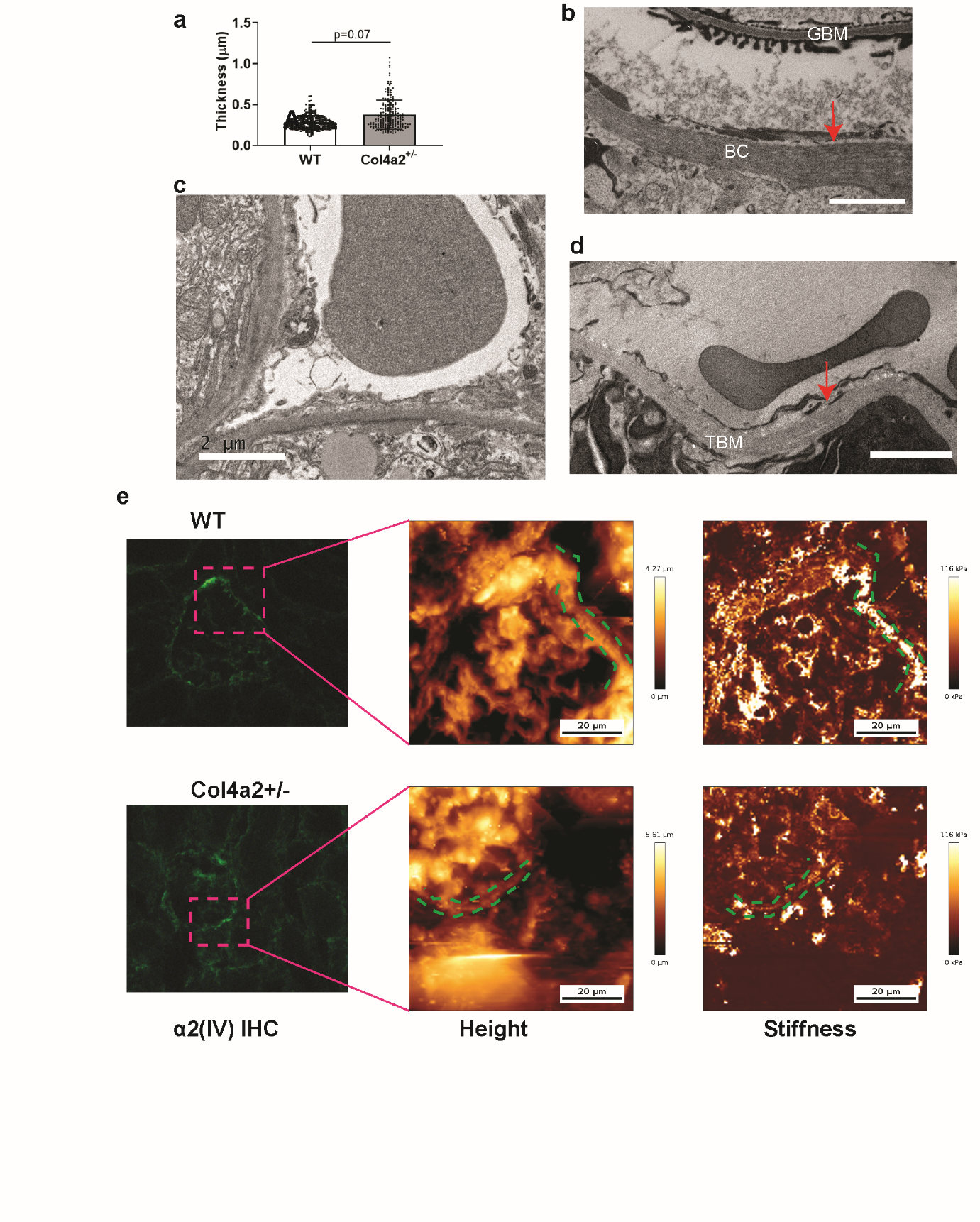
**

**Supplemental Figure 3: Basement membrane defects in *Col4a2^+/em2Wtsi^* mice.** (a) Thickness of kidney tubular basement membrane. Data points are individual measures from n=3 mice. p=0.07, Welch’s t-test on n=3 ,point estimate (95% CI -0.01841 to 0.2285), p<0.001 vs WT cumulative distribution analysed by Kolmogorov-Smirnov test total measurement: *Col4a2^+/em2Wtsi^* n=204; WT n= 299 (b) Normal regular appearance of glomerular basement membrane (GBM) but thickening of BM of Bowmans Capsule (BC) with evidence of splitting into multiple strands (red arrows). (c) Vascular and tubular BM with appearance of an irregular diffuse BM with thickening of two strands (red arrow) of BM. (d) BM Splitting and strand formation (red arrow) in tubular BM (TBM) and vascular basement membrane. Size bar 2µm. (e) Atomic Force Microscopy of Bowman’s Capsule***.*** Staining against α2(IV) (green) showing BM of Bowman’s Capsule was used to guide AFM analysis in wild type (WT) and *Col4a2^+/em2Wtsi^*  (Col4a2^+/-^). Magenta box highlight area depicted in middle and right-hand panel. Middle panel shows AFM image of height of Bowmans Capsule (green dotted line). Right-hand panel AFM image of stiffness of Bowmans Capsule (green dotted line). The AFM image of the stiffness is the same image as provided in Figure 2g..

**Supplemental Figure 4**

**
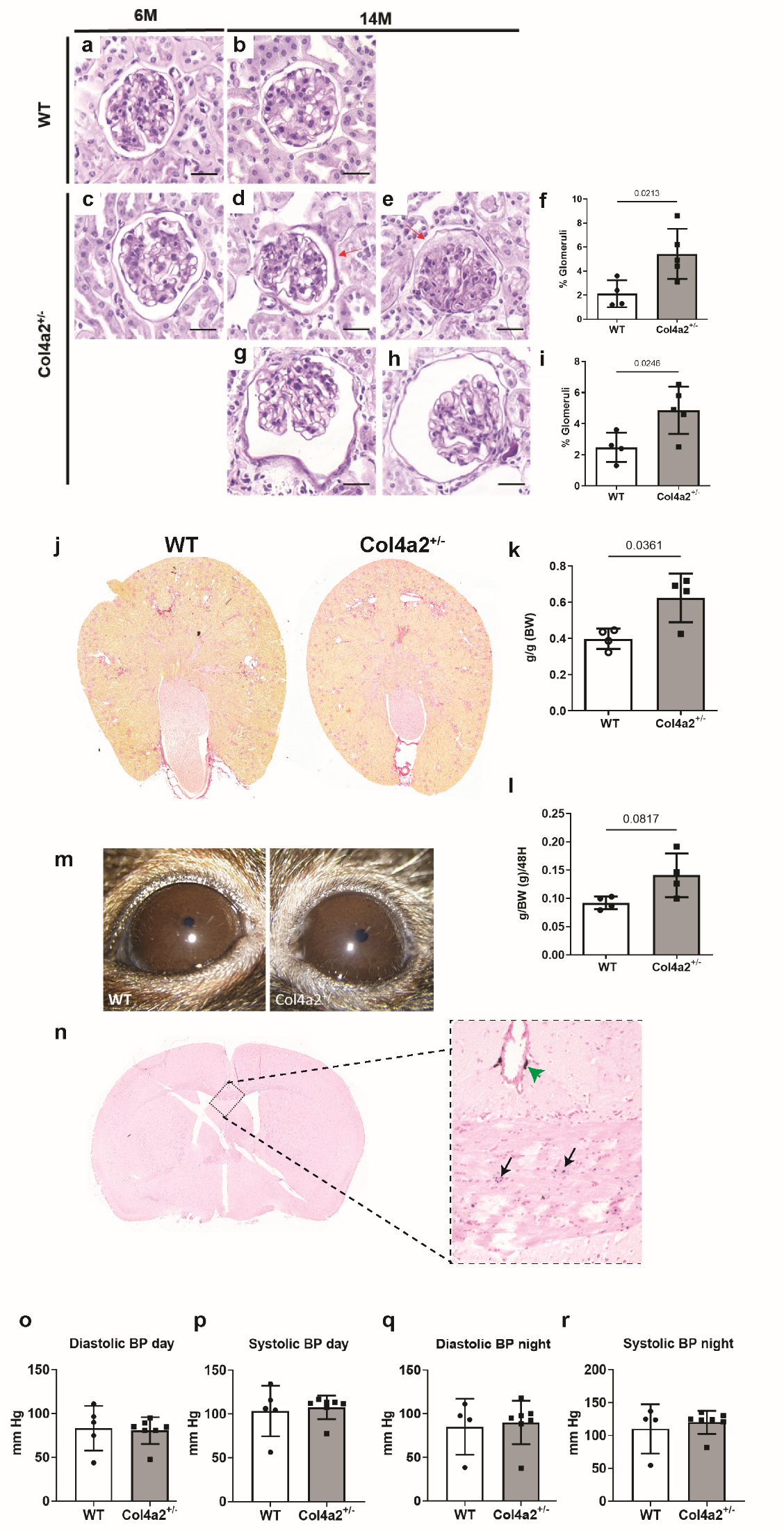
**

**Supplemental Figure 4: Phenotype analysis *Col4a2^+/em2Wtsi^* mice.** (a-e, g-h) Histopathology of kidney sections of 6 (a, c) and 14-month-old mice (b, d-e, g-h). 14 month old *Col4a2^+/em2Wtsi^* (Col4a2^+/-^) display thickening of Bowmans capsule zone with splitting (red arrow). (f) Percentage glomeruli showing Bowman’s Capsule thickening in 14-month-old mice. Point estimate 3.315 (95%CI 0.6822 to 5.948) (g-h) Capillary tuft retraction in *Col4a2^+/em2Wtsi^* . (I) Percentage of glomeruli showing capillary tuft retraction in 14-month-old mice. Point estimate 2.385 (95%CI 0.4136 to 4.356) (a-i n= 5 mice; * p<0.05, ** p<0.01, Welch’s t-test). (j) Picrosirius Red staining reveals no overt fibrosis. (k) Water consumption per gram bodyweight over 2 day period in 14 month old WT and *Col4a2^+/em2Wtsi^* mice. Welch’s t-test n=4, point estimate 0.2258 (95% CI 0.02376 to 0.4279). (l) Urinary production per gram bodyweight in 14 month old WT and *Col4a2^+/em2Wtsi^* over 48h. Welch’s t-test, n=4, point estimate 0.0488 (95% CI -0.01045 to 0.1082). (m) Slit lamp analysis of 14 month old mice shows no overt anterior segment defects. (n=5) (n) Hemosiderin positive staining (black arrow) and brown potential lipofuscin stain (green arrowhead) in brain of 14 month old *Col4a2**^+/em2Wtsi^* mice. (o) Daytime diastolic blood pressure of 4-month old WT and *Col4a2^+/em2Wtsi^* mice (p) Daytime systolic blood pressure (q) Nighttime diastolic blood pressure (r) Nighttime systolic blood pressure. (o-r Mann-Whitney U test)

**Supplemental Figure 5**

**
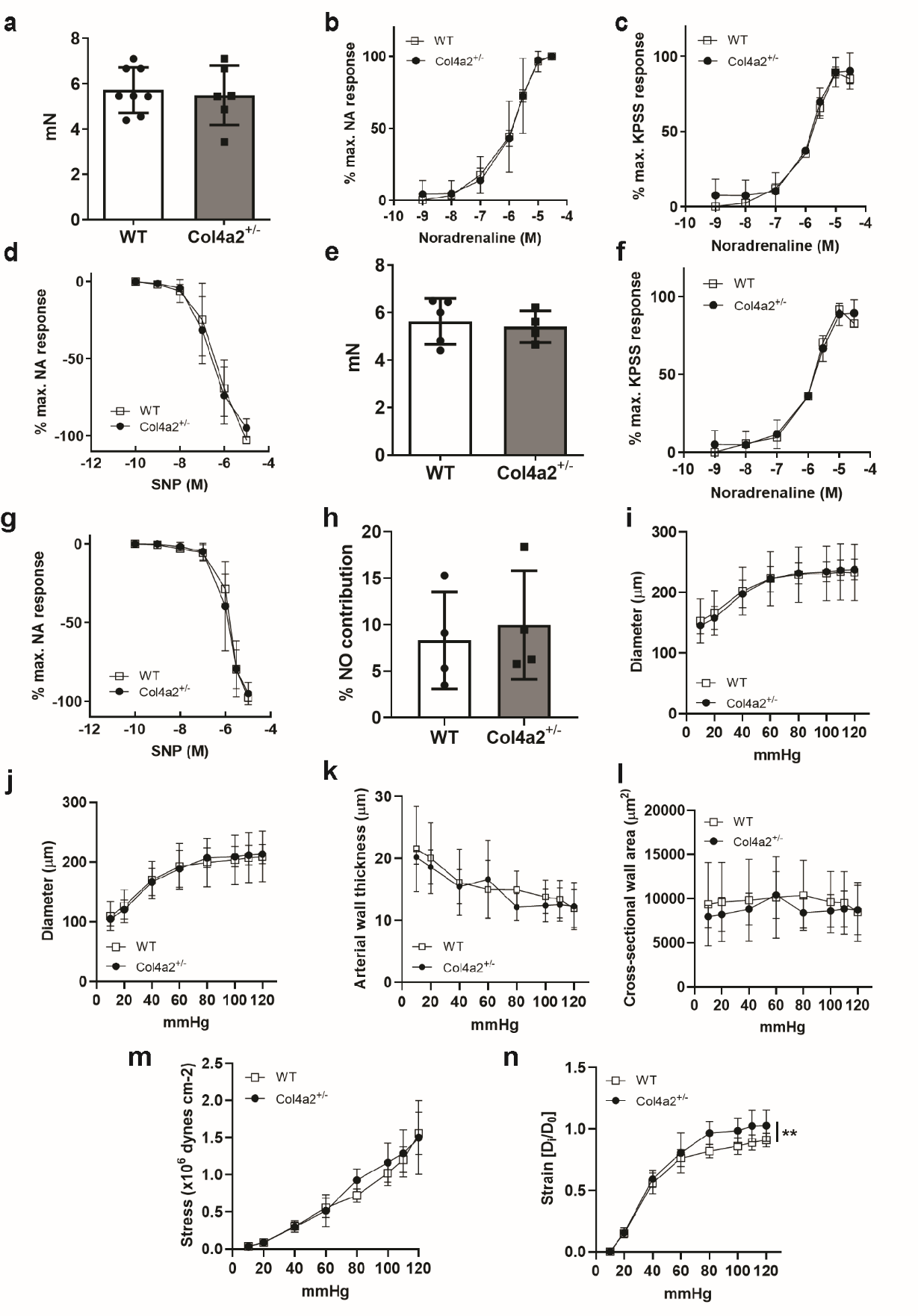
**

**Supplemental Figure 5. Vascular function and structure in *Col4a2^+/em2Wtsi^* mice.** (a) Total constriction to KPSS of three-month-old wildtype (WT) and *Col4a2^+/em2Wtsi^* mice (Col4a2^+/-^). (Welch’s t-test, n=5) (b) Vasoconstriction to noradrenaline in three-month-old and *Col4a2^+/em2Wtsi^* mice (n=5, area under the curve and Welch’s t-test). (c) Noradrenaline vasoconstriction expressed as percentage of response to KPSS in three-month-old and *Col4a2^+/em2Wtsi^* mice. (n=5, area under the curve and Welch’s t-test) (d) Endothelial cell independent vasodilation measured by dose response curve to SNP in three-month-old WT and *Col4a2^+/em2Wtsi^* mice. (n=5; area under the curve and Welch’s t-test). (e) Maximum vasoconstriction to KPSS in 6-month-old WT and *Col4a2^+/em2Wtsi^* mice (n=4-5, Welch’s t-test). (f) Vasoconstriction to Nor-adrenaline dose relative to maximum response to KPSS in 6-month-old WT and *Col4a2^+/em2Wtsi^* mice (n=4-5, area under the curve and Welch’s t-test). (g) Endothelial cell independent vasodilation assessed by dose response to SNP of vessels pre-constricted with noradrenaline in 6-month-old WT and *Col4a2^+/em2Wtsi^* mice. (n=4-5, area under the curve and Welch’s t-test). (h) Basal NO generation in 6-month-old WT and *Col4a2^+/em2Wtsi^* mice measured indirectly by wire myography. Arteries were subjected to 10 μM noradrenaline with and without pre-treatment of LNAME. Difference in level of constriction presented as the relative contribution of basal NO (n=4-5, Welch’s t-test). (i) Outer diameter of mesenteric arteries over range of pressures in 6-month-old WT and *Col4a2^+/em2Wtsi^* mice (area under the curve followed by unpaired t-test, n=5). (j) Inner diameter of mesenteric arteries of 6-month-old WT and *Col4a2^+/em2Wtsi^* mice (area under the curve followed by Welch’s t-test, n=5). (k) Arterial wall thickness of 6-month-old WT and *Col4a2^+/em2Wtsi^* mice (area under the curve followed by Welch’s t-test, n=5). (l) Cross sectional wall area of 6-month-old WT and *Col4a2^+/em2Wtsi^* mice (area under the curve followed by Welch’s t-test, n=5). (m) Stress of vascular wall in 6-month-old WT and *Col4a2^+/em2Wtsi^*. (area under the curve followed by Welch’s t-test; n=5) (n) Strain of vascular wall in 6-month-old WT and *Col4a2^+/em2Wtsi^.* (area under the curve followed by Welch’s t-test, n=5, point estimate 8.388 (95% CI 4.347 to 12.43)) ****** p<0.01

**Supplemental Figure 6**

**
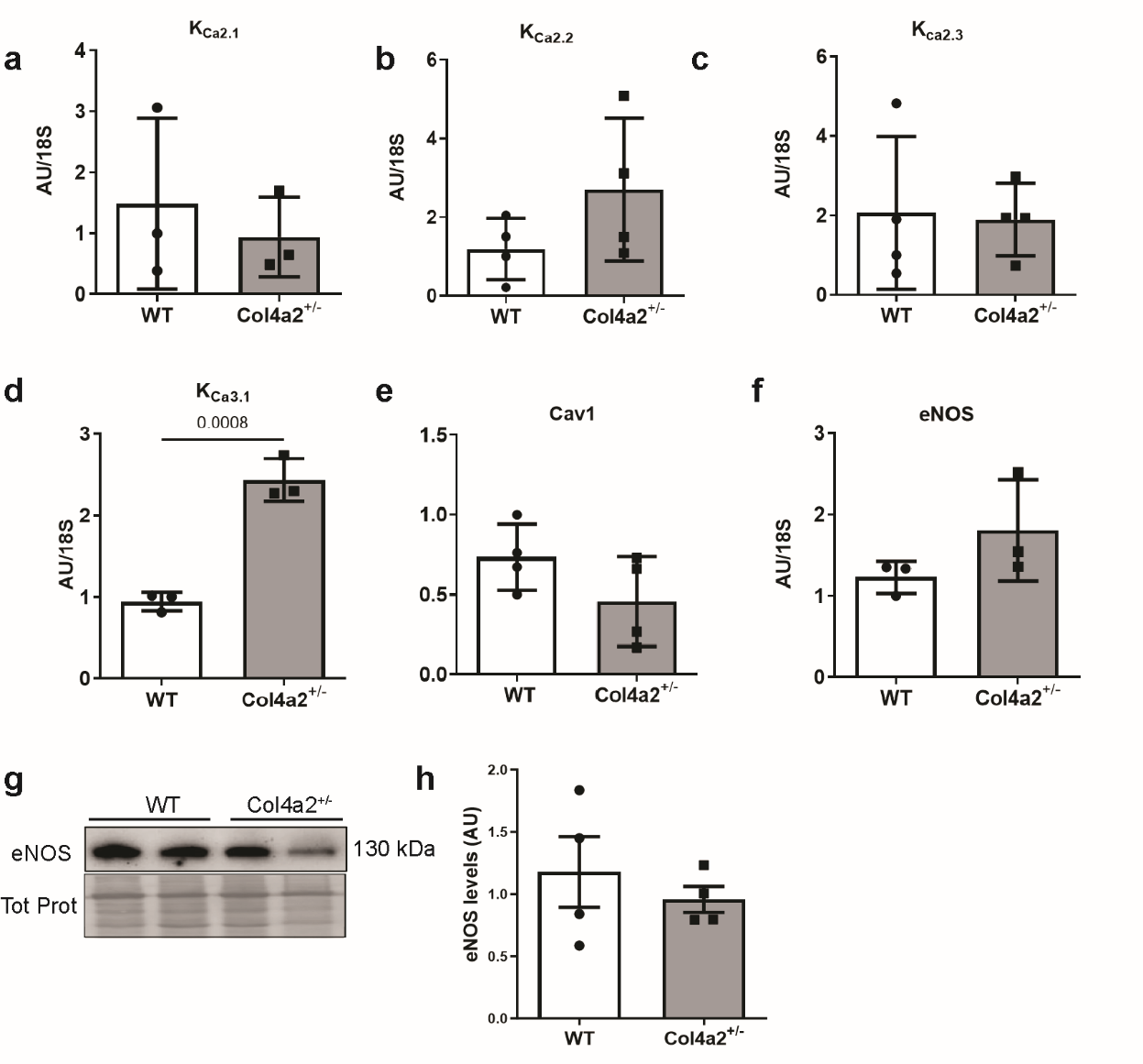
**

**Supplemental Figure 6. Analysis of EDH-related mRNA and protein levels in *Col4a2^+/em2Wtsi^* mice.** (a-c) mRNA levels of small K_Ca_ channels K_Ca2.1-2.3_ in mesentery of 6 month old wild type (WT) and *Col4a2^+/em2Wtsi^* mice (Col4a2) (d) Increased mRNA levels of *KCNN4*, encoding the intermediate K_Ca3.1_ channel. Point estimate 1.492 (95% CI 0.9361 to 2.048). (e) Caveolin 1 (*Cav1*) mRNA levels (f) *eNOS* mRNA levels. Levels are normalized to 18S rRNA. (g-h) Western blotting against eNOS (using Ponceau stain [Tot Prot] as total protein loading control. Western blotting against n=3-4, Welch’s t-test.

**Supplemental Figure 7**

**
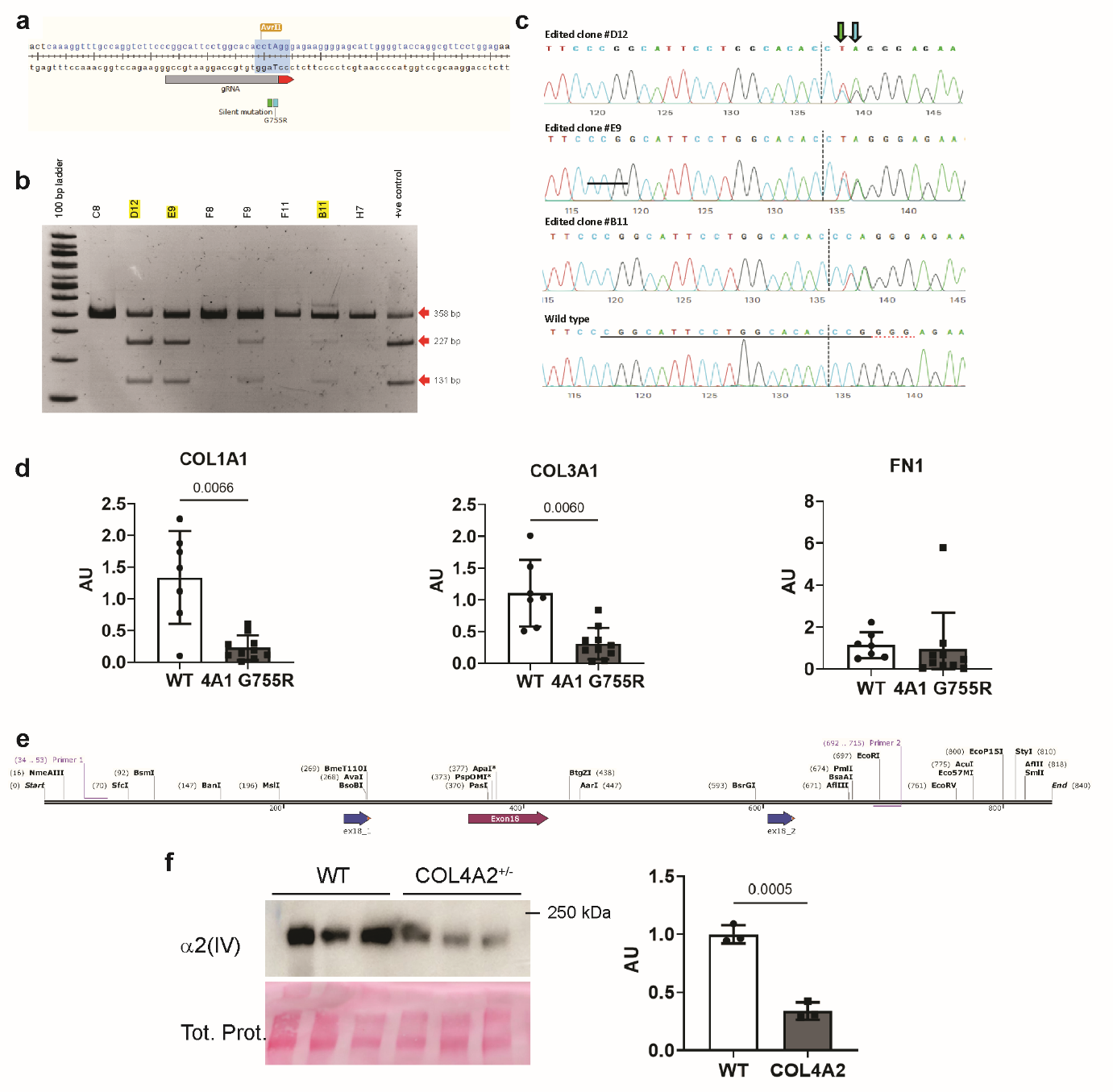
**

**Supplemental Figure 7.** ***COL4A1* G755R endothelial cells**. (a) Schematic diagram showing the site of *COL4A1* modification. Donor template (blue font) carries the *COL4A1* G755R (c.2263G->A, capital) and a silent mutation (green, CCCAGG→CCTAGG) to incorporate the AvrII recognition site for screening. PAM site (red); sgRNA (grey). (b) Single cell-derived gene-edited clones are indicated by the presence of AvrII restriction bands. (c) Sequence analysis of edited clones. Guide RNA is underlined. Blue arrow:G755R mutation, Green arrow: silent variant for genotyping. (d) qRT-PCR analysis did not provide evidence of a synthetic phenotype in COL4A1^G755R/+^ cells. *COL1A1* (collagen I alpha chain 1) point estimate -1.106 (95% CI -1.617 to -0.5947), *COL3A1* (collagen III alpha chain 1) point estimate -0.795 (95% CI -1.199 to -0.3919), *FN1* (fibronectin 1). WT (wild-type, n=7), COL4A1 (*COL4A1^G755R/+^*, n=10) Welch’s t-test (e) Diagram showing CRSIPR guides to delete exon 18 of COL4A2 in HBEC. (f) Western blot against α2(IV) encoded by COL4A2 on conditioned media of COL4A2^+/-^ cells and wild type cells (WT) (n=3 Welch’s t-test point estimate -0.662 (95%CI -0.8383 to -0.4858)). (a) and (f) were generated using Snapgene software.

**Supplemental Figure 8**


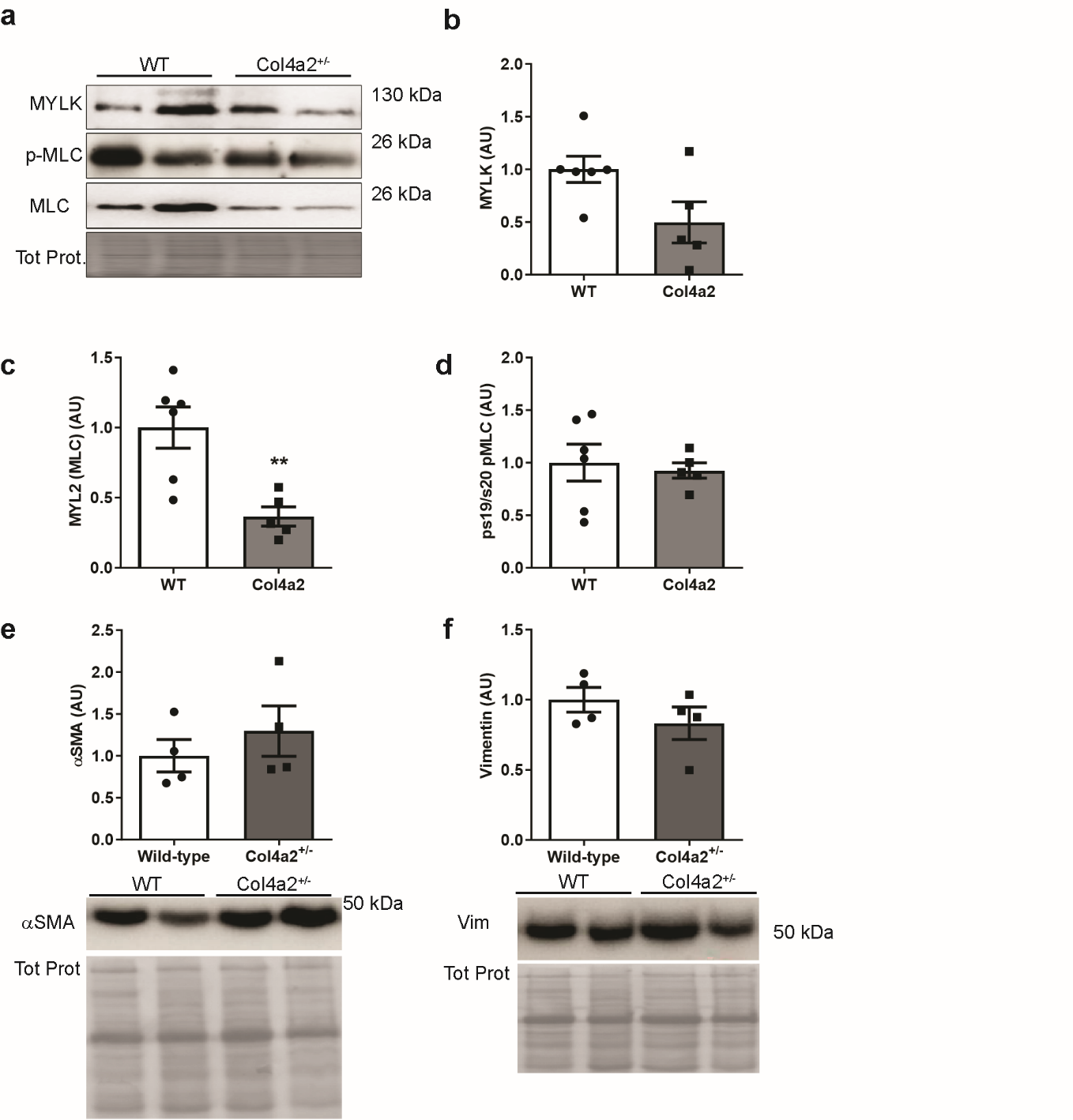


**Supplemental Figure 8.** **Calcium related protein levels and vascular smooth muscle cell markers in *Col4a2^+/em2Wtsi^***. Western blotting of protein lysates from mesenteric vasculature of 6-month-old mice. (a) Duplication of Figure 4c. Western blotting against myosin light chain kinase (MYLK), total myosin light chain (MLC) and phosphorylated MLC (p-MLC). Tot. prot: Ponceau stain of total protein loading. (b-d) Quantification of data depicted in (a). (e) Western blotting against α smooth muscle actin (αSMA). (f) Western blotting against vimentin. Tot. prot: Ponceau stain of total protein loading.

**Supplemental Figure 9**

**
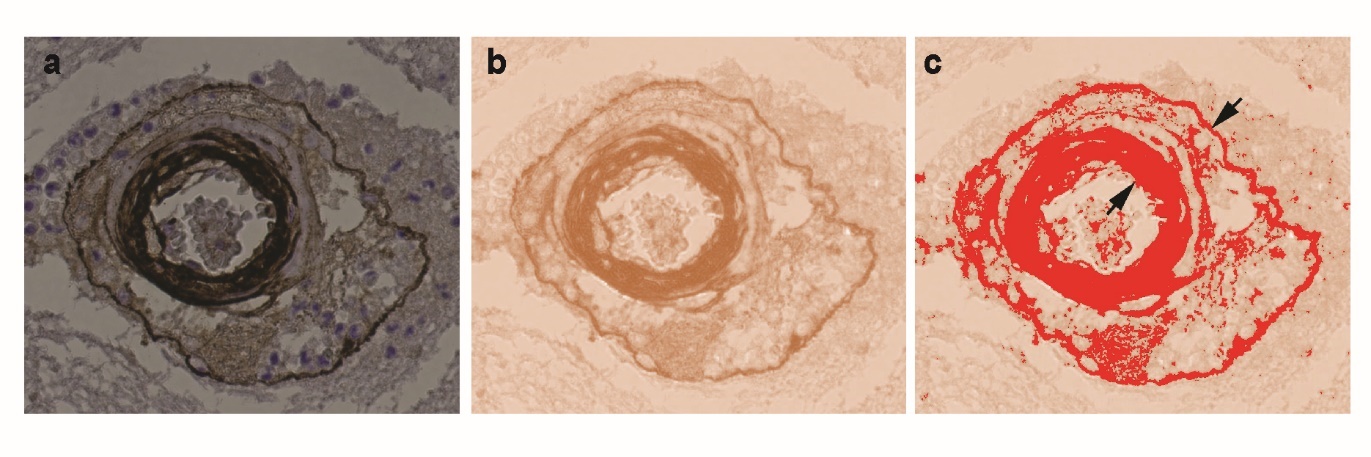
**

**Supplemental Figure 9. Image analysis of vascular collagen IV in human brain tissue.** Example of measurement of vascular collagen IV labelled area. Raw images (a, 20 x magnification, duplication of image in Figure 5a) are divided into colour channels using automated plug-in (ImageJ; H DAB) (B). Pixels labelled for collagen-IV (brown, b) are detected by an algorithm (ImageJ; auto-threshold, **c**). Arrows indicate endothelial and parenchymal basement membranes that were used to measure vascular wall thickness.

**Supplemental Table 1: Demographics and clinical data of patient cohort.**

|  | **Total Cohort** | **Controls** | **SVD** | **COL4-ICH** |
| --- | --- | --- | --- | --- |
| n | 43 | 18 | 18 | 7 |
| Male % (n)  Female % (n) | 51% (22)  49% (21) | 56% (10)  44% (8) | 44% (8)  56% (10) | 57% (4)  43% (3) |
| Age (years), (S.D) | 68.9 (17.9) | 49.7 (9.1) | 83.9 (5.6) | 79.7 (5.3) |
| Selection Criteria |  | Non-ICH, non-traumatic brain injury, absence of rare coding variants. | Sporadic SVD with high SVD score (2 or 3), positive for deep ICH, absence of rare coding variants in COL4A1/2 | Sporadic ICH with rare coding variants in COL4A1/ |
| Ethnicity |  | Data not collected | 16/18 White British (89%)  1/18 black British (5.5%)  1/18 no data (5.5%) | 7/7 White British (100%) |
| Smoking  Never  Ex  Current  No data |  | 33% (6)  11% (2)  44% (9)  5.5% (1) | 56% (10)  33% (6)  5.5% (1)  5.5% (1) | 57% (4)  43% (3) |
| Diabetes  No  Type 2 |  | 89% (16/18)  11% (2/18) | 89% (16)  11% (2) | 86% (6)  13% (1) |
| Hypertension  Yes  No  No data |  | 11% (2)  89% (16) | 89% (16)  5.5% (1)  5.5% (1) | 57% (4)  43% (3) |

**Supplemental Table 2: Primers and antibody details**

| **Gene** | **Primer F (5’-3’)** | **Primer R (5’-3’)** | | |
| --- | --- | --- | --- | --- |
| *Col4a1* | AGCGCCAACAAATTGGGGA | TTCACGCCATGACAGTCACA | | |
| *Col4a2* | AAGGGATCGAGCCGAGTCTA | GAGGGCCAGACGCTTTGAA | | |
| *eNOS* | GTTTCCAAGGAAGTTACAGAGCC | GGGAGCCACTCCTTTTGATG | | |
| *SK1* (small K_Ca 2.1_) | AACTTCCTGGGAGCCATGTG | TGAGCAGACACACACCCTTC | | |
| *SK2* (small K_Ca 2.2_) | ACTAGCAACTTCCTTGGAGCA | TCCAGTAAGCAAGCAGACTCC | | |
| *SK3* (small K_Ca 2.3_) | TGCAGATGACTGGCGGATAG | CTTGTACTCTCCAGGGATGGG | | |
| *Cav1* (caveolin 1) | AGCATGTCTGGGGGCAAATAC | CTTGACCACGTCGTCGTTGAG | | |
| *KCNN4* (intermediate K_Ca 3.1_) | GCTCCTGCGTCTCTACCTG | GAAGCGGACTTGGTTGAGC | | |
| *COL1A1* | AGTGGTTTGGATGGTGCCAA | GCACCATCATTTCCACGAGC | | |
| *COL3A1* | TGGTCTGCAGGAATGCCTGGA | TCTTTCCCTGGGACACCATCAG | | |
| *FN1* | CTGGGTCTCCTCCCAGAGAA | GGGTGTGGAAGGGTTACCAG | | |
| **Protein** | | **Supplier and Catalogue Number** | | **Dilution** |
| Bip | | BD Transduction #610979; RRID:AB_398292 | | 1/40,000 WB |
| eIF2α | | Cell Signaling Technology #9722; RRID:AB_2230924 | | 1/1000 WB |
| p-eIF2α | | Cell Signaling Technology #9721; RRID:AB_330951 | | 1/1000 WB |
| Grp94 | | Santa Cruz Technology #sc-32249; RRID:AB_627676 | | 1/1000 WB |
| Col4a1 | | Gifted Dr. Yasuko Tomono H11, CHONDREX Cat# 7070, RRID:AB_2904114 | | 1/70 IHC |
| Col4a2 | | Gifted Dr. Yasuko Tomono H22; CHONDREX Cat# 7071, RRID:AB_2904115 | | 1/70 IHC |
| αSMA | | Abcam #ab7817; RRID:AB_262054 | | 1/1000 WB |
| Vimentin | | Proteintech #10366-1-AP; RRID:AB_2273020 | | 1/10,000 WB |
| KCNN4 (Kca_3.1_) | | Proteintech #23271-1-AP; RRID:AB_2879243 | | 1/4000 WB |
| eNOS | | Celll Signalling Technology #9572; RRID:AB_329863 | | 1/1000 WB |
| MYLK | | Proteintech #21642-1-AP; RRID:AB_10734590 | | 1/500 WB |
| MLC-2V | | Proteintech #21642-1-AP; RRID:AB_10734590 | | 1/500 WB |
| Myosin pS19/S20 | | Rockland #600-401-416S; RRID:AB_11182907 | | 1/500 WB |
| Anti-rat IgG | | Alexa Fluor 568 ThermoFisher | | 1/300 IHC |
| Anti-Rabbit IgG HRP | | Cell Signalling Technology #7074S | | 1/1000 WB |
| m-IgG Fc BP-HRP | | Santa Cruz Biotechnology #sc-525416 | | 1/1000 WB |

WB: western blot. IHC: immunohistochemistry

**References**

1. Reissig LF, Herdina AN, Rose J, Maurer-Gesek B, Lane JL, Prin F, et al. The Col4a2(em1(IMPC)Wtsi) mouse line: lessons from the Deciphering the Mechanisms of Developmental Disorders program. Biol Open. 2019;8(8).
