# Supplementary material for: Collagen IV deficiency causes hypertrophic remodeling and endothelium-dependent hyperpolarization in small vessel disease with intracerebral hemorrhage
Source: eBioMedicine. 2024 Aug 30;107:105315. doi: 10.1016/j.ebiom.2024.105315 (PMC11402910; doi:10.1016/j.ebiom.2024.105315)
Supplement: Western Blots [file mmc2.docx]

Western Blots


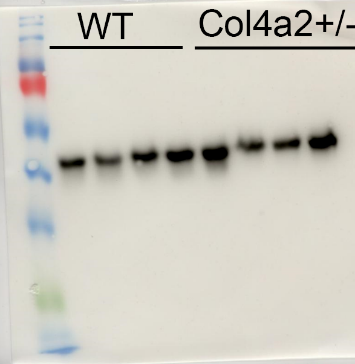


Figure 3f KCNN4


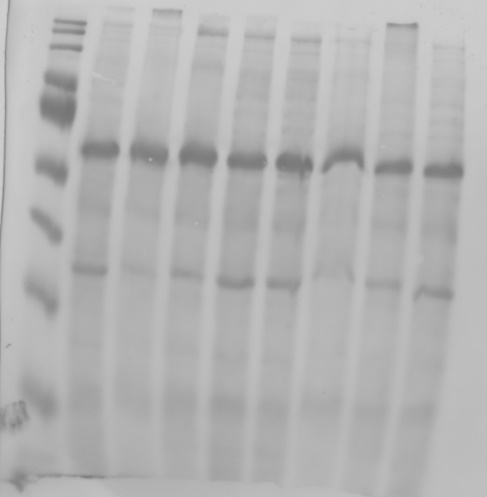


Figure 3f Ponceau Total Protein Stain


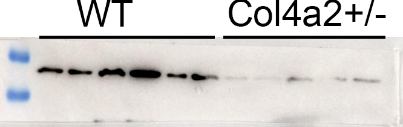


Figure 4i MLC


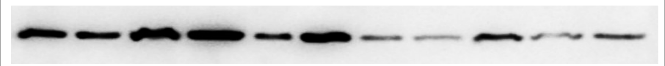


Figure 4i MLC exposure used in figure


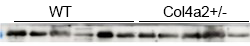


Figure 4i p-MLC


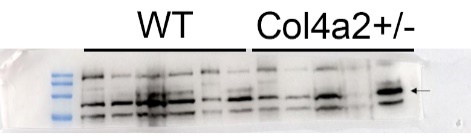


Figure 4i MYLK


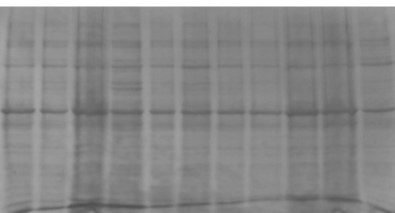


Figure 4i Ponceau Total Protein Stain


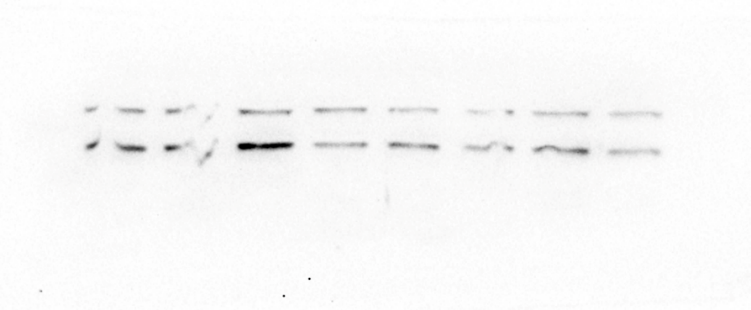


Supplemental Figure 2l eif2p


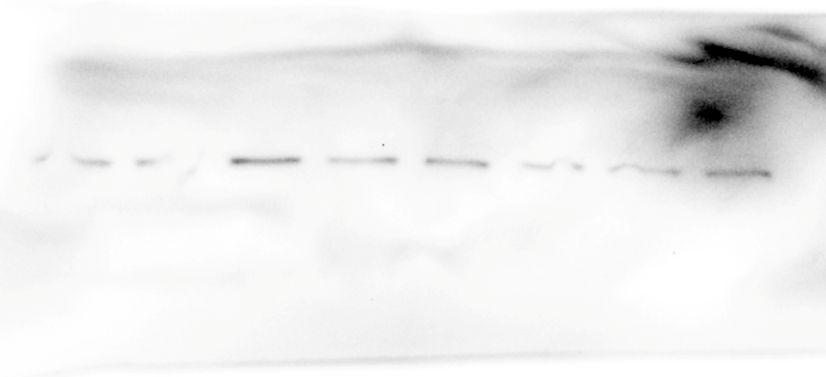


Supplemental Figure 2l eif2


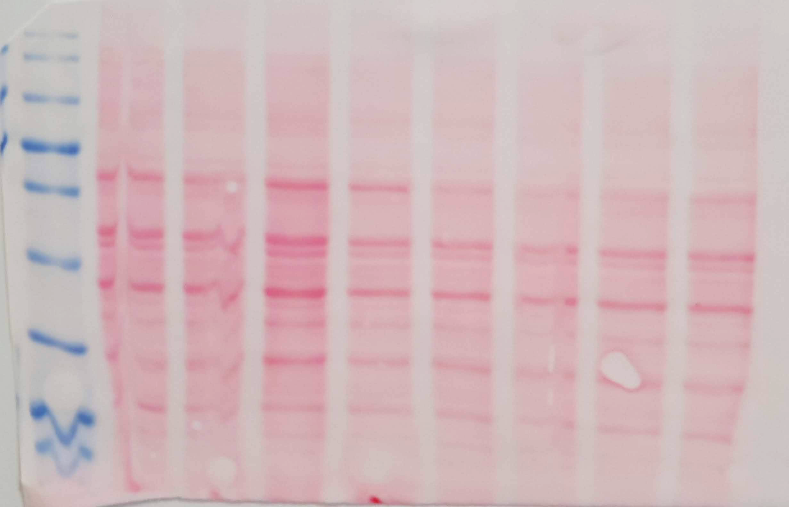


Supplemental Figure 2l Total Protein Stain


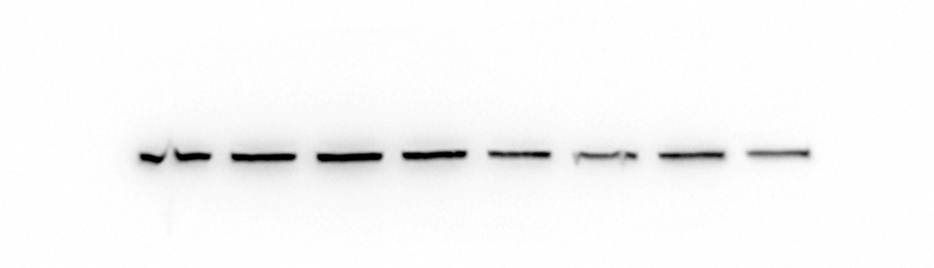
Supplemental Figure 2m Grp94


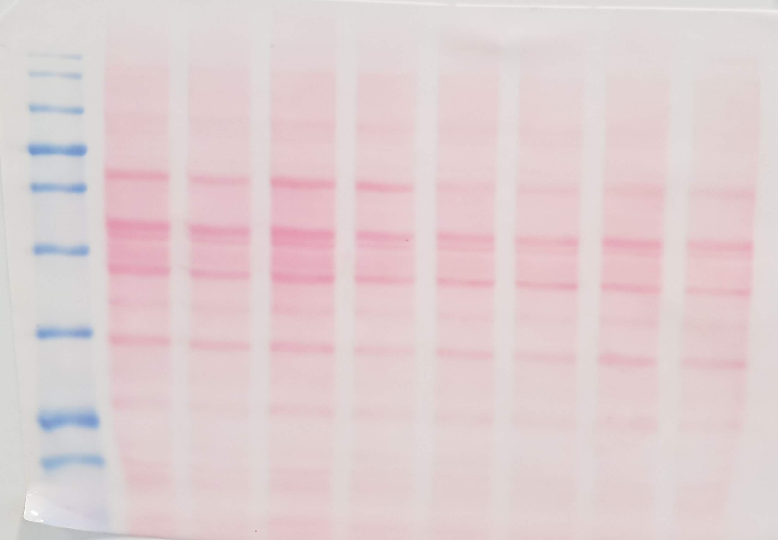


Supplemental Figure 2m Ponceau Total Protein Stain


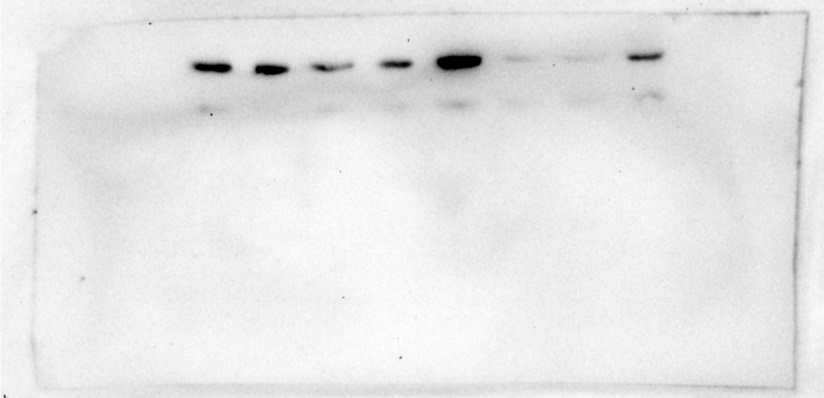


Supplemental Figure 2n eif2P


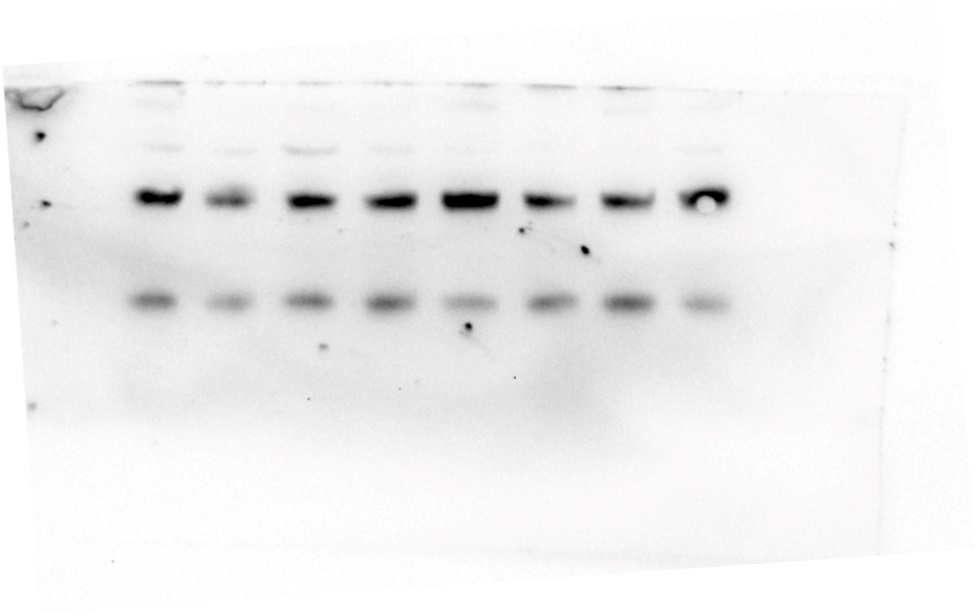


Supplemental Figure 2n eif2


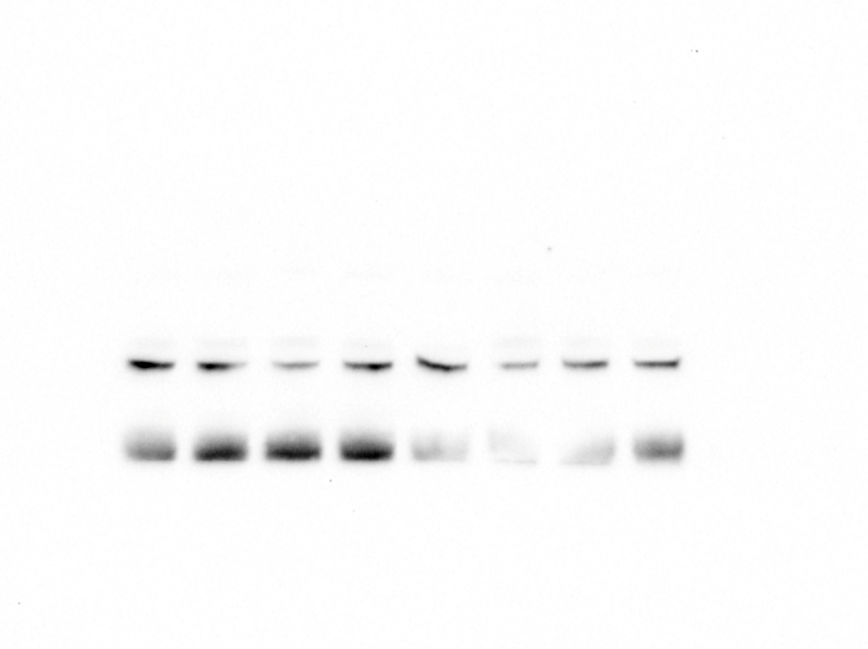
Supplemental Figure 2n Bip


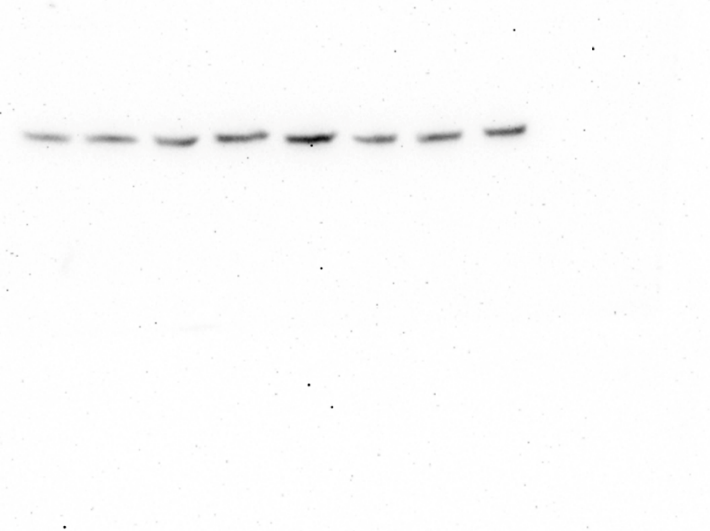


Supplemental Figure 2n VDAC


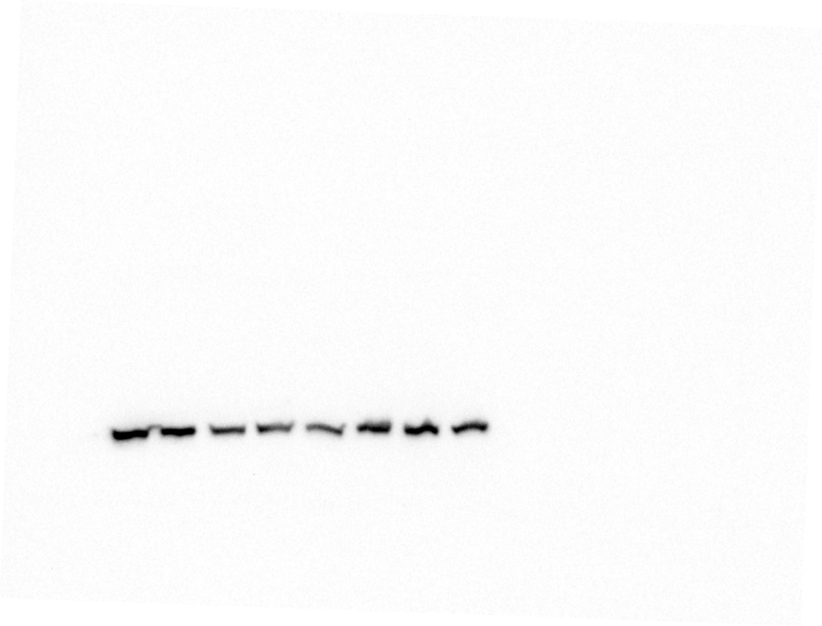


Supplemental Figure 2o Grp94


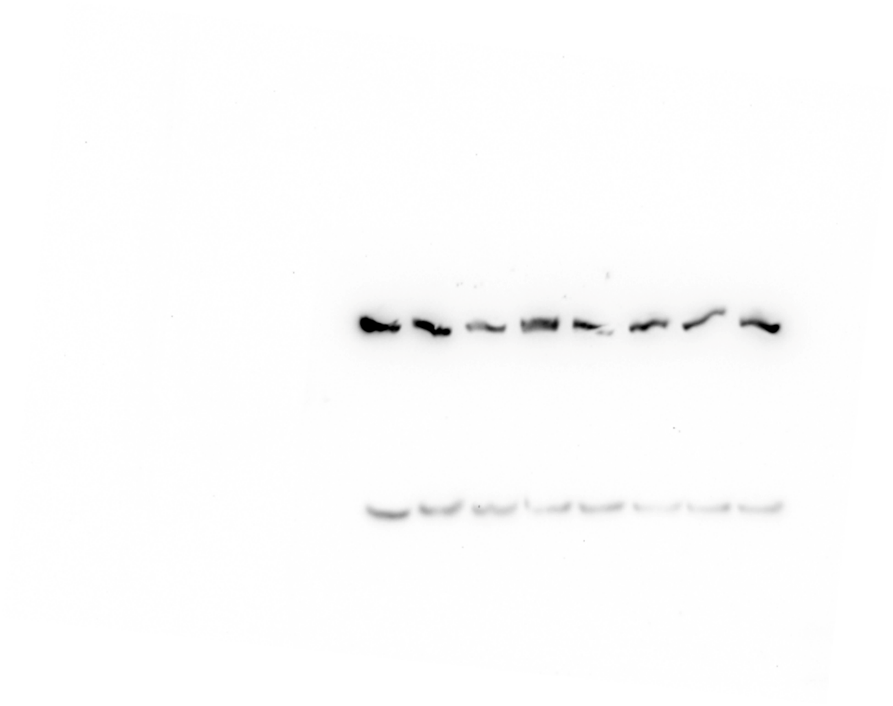


Supplemental Figure 2o VDAC (bottom band)


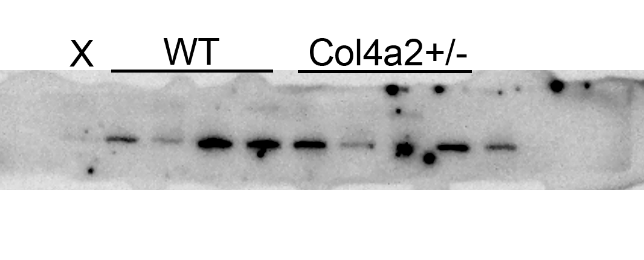


Supplemental Figure 6g eNOS. Lanes marked X were not part of the investigation.


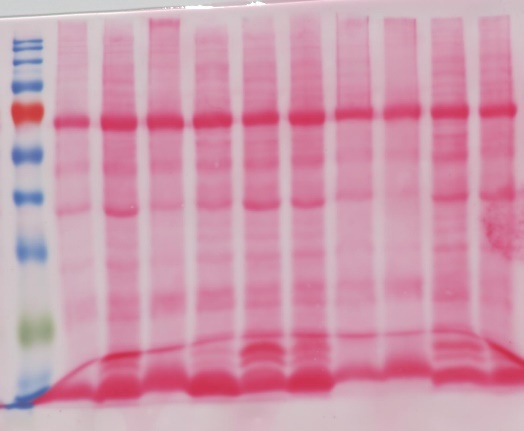


Supplemental Figure 6g Ponceau Total Protein Stain


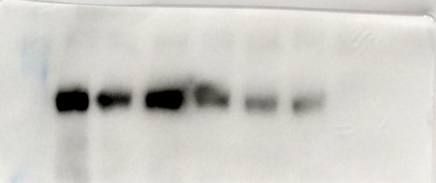


Supplemental Figure 7f COL4A2


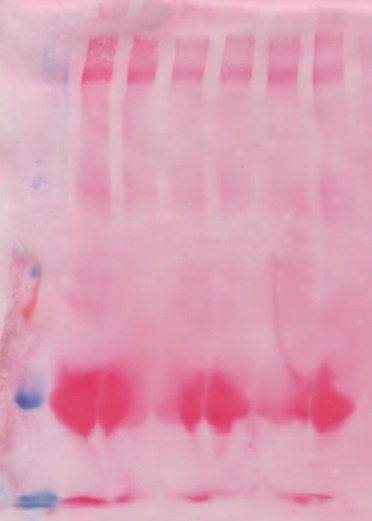


Supplemental Figure 7f Ponceau Total Protein Stain


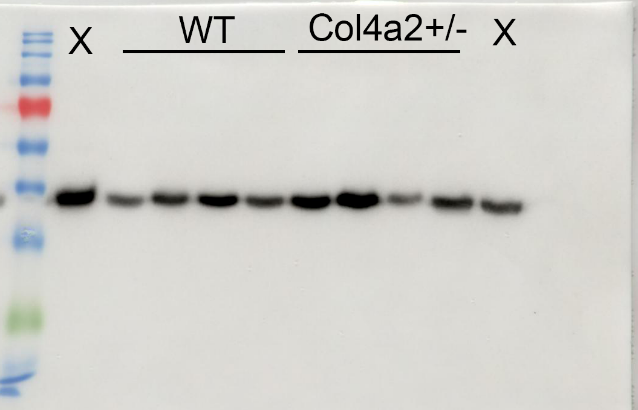


Supplemental Figure 8e αSMA X lanes were not part of the investigation


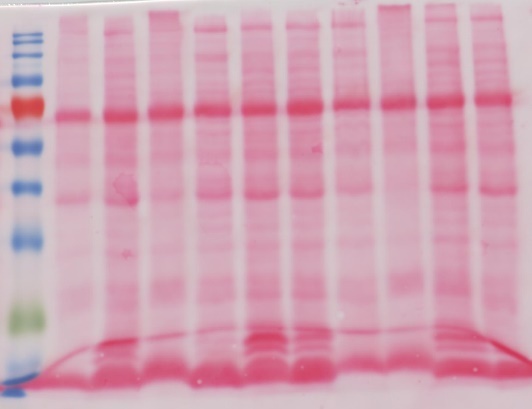


Supplemental Figure 8e Ponceau Total Protein Stain


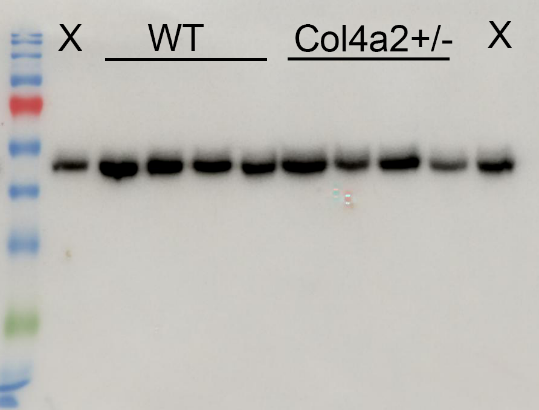


Supplemental Figure 8f Vimentin. Lanes marked X were not part of the investigation.


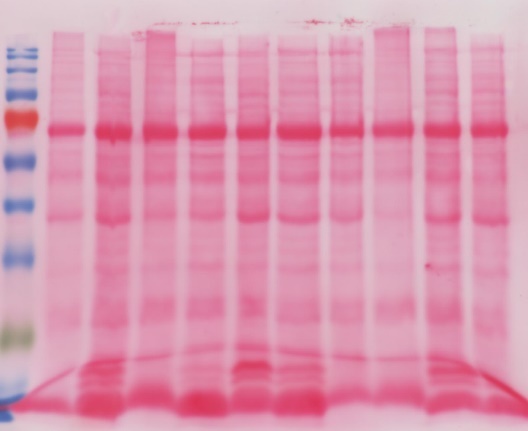


Supplemental Figure 8f Ponceau Total Protein Stain
